# Low-Cost Hourly Ambient Black Carbon Measurements at Multiple Cities in Africa

**DOI:** 10.1021/acs.est.4c02297

**Published:** 2024-07-02

**Authors:** Abhishek Anand, N’Datchoh Evelyne Touré, Julien Bahino, Sylvain Gnamien, Allison Felix Hughes, Raphael E Arku, Victoria Owusu Tawiah, Araya Asfaw, Tesfaye Mamo, Sina Hasheminassab, Solomon Bililign, Vaios Moschos, Daniel M. Westervelt, Albert A. Presto

**Affiliations:** † Center for Atmospheric Particle Studies, Carnegie Mellon University, Pittsburgh, Pennsylvania 15213, United States; ‡ Department of Mechanical Engineering, Carnegie Mellon University, Pittsburgh, Pennsylvania 15213, United States; § Université Félix Houphouët-Boigny, Abidjan 00225, Côte d’Ivoire; ∥ University of Ghana, Accra 00233, Ghana; ⊥ Department of Environmental Health Sciences, University of Massachusetts Amherst, Amherst, Massachusetts 01003, United States; # Department of Meteorology & Climate Science, Kwame Nkrumah University of Science and Technology, Kumasi 00233, Ghana; ∇ Institute of Geophysics, Space Science and Astronomy, Addis Ababa University, Addis Ababa 1176, Ethiopia; ○ Jet Propulsion Laboratory, California Institute of Technology institution, Pasadena, California 91011, United States; ◆ Department of Physics, North Carolina A&T State University, Greensboro, North Carolina 27411, United States; ¶ Lamont Doherty Earth Observatory, Columbia University, New York, New York 10964, United States

**Keywords:** atmospheric black carbon, beta attenuation monitors, image processing, hourly measurements, low-cost monitoring, sub-Saharan Africa

## Abstract

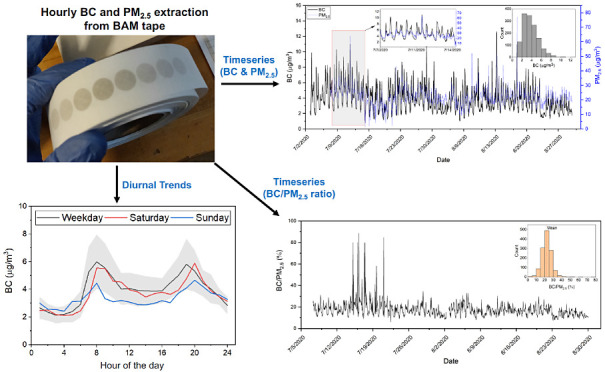

There is a notable lack of continuous monitoring of air pollutants in the Global South, especially for measuring chemical composition, due to the high cost of regulatory monitors. Using our previously developed low-cost method to quantify black carbon (BC) in fine particulate matter (PM_2.5_) by analyzing reflected red light from ambient particle deposits on glass fiber filters, we estimated hourly ambient BC concentrations with filter tapes from beta attenuation monitors (BAMs). BC measurements obtained through this method were validated against a reference aethalometer between August 2 and 23, 2023 in Addis Ababa, Ethiopia, demonstrating a very strong agreement (*R*^2^ = 0.95 and slope = 0.97). We present hourly BC for three cities in sub-Saharan Africa (SSA) and one in North America: Abidjan (Côte d’Ivoire), Accra (Ghana), Addis Ababa (Ethiopia), and Pittsburgh (USA). The average BC concentrations for the measurement period at the Abidjan, Accra, Addis Ababa Central summer, Addis Ababa Central winter, Addis Ababa Jacros winter, and Pittsburgh sites were 3.85 μg/m^3^, 5.33 μg/m^3^, 5.63 μg/m^3^, 3.89 μg/m^3^, 9.14 μg/m^3^, and 0.52 μg/m^3^, respectively. BC made up 14–20% of PM_2.5_ mass in the SSA cities compared to only 5.6% in Pittsburgh. The hourly BC data at all sites (SSA and North America) show a pronounced diurnal pattern with prominent peaks during the morning and evening rush hours on workdays. A comparison between our measurements and the Goddard Earth Observing System Composition Forecast (GEOS-CF) estimates shows that the model performs well in predicting PM_2.5_ for most sites but struggles to predict BC at an hourly resolution. Adding more ground measurements could help evaluate and improve the performance of chemical transport models. Our method can potentially use existing BAM networks, such as BAMs at U.S. Embassies around the globe, to measure hourly BC concentrations. The PM_2.5_ composition data, thus acquired, can be crucial in identifying emission sources and help in effective policymaking in SSA.

## Abstract 2

This study applied a novel low-cost method to quantify hourly ambient BC and BC:PM_2.5_ using an existing network of regulatory PM_2.5_ monitors in sub-Saharan Africa. This method can aid evidence-based policymaking to mitigate air pollution in developing nations.

## 1 Introduction

Exposure to air pollution poses a severe risk to global public health.^1−3^ The risk is more pronounced in low- and middle-income countries (LMICs), including in cities in Africa, due to high exposure levels resulting from rapid urban and economic growth. An estimated 1.2 million deaths out of the global 5.5 million premature deaths from air pollution occur in Africa annually.^4−6^ Fine particulate matter (PM_2.5_) pollution is the primary driver of exposure risk.^7^

Black carbon (BC) is one of the major components of PM_2.5_. There is suggestive evidence that BC poses higher specific toxicity than overall PM_2.5_.^8,9^ The contribution of BC to PM_2.5_ varies by local environments because of differences in sources.^10^ More than 4.4 billion people globally live in urban environments^11^ and are exposed to BC primarily from the combustion of fossil fuels in vehicles and power plants.^12^ Rural populations in developing nations may be exposed to high BC concentrations due to agricultural burning and solid fuel use for cooking and heating purposes.^13^

BC is a short-lived climate-forcing agent. It strongly absorbs incoming solar radiation across the spectral band including ultraviolet, visible, and infrared wavelengths, consequently adding to the absorbed solar radiation by the earth-atmosphere system.^14^ Thus, BC significantly adds to the global radiative forcing, which imbalances the earth’s radiation budget.

Worldwide, there is a high disparity in the number, density, and chemical specificity of ground monitors measuring air pollutants. Air quality in the U.S. is regulated by the Environmental Protection Agency (EPA) under the Clean Air Act, and there is a comprehensive infrastructure to measure pollutant concentrations nationwide. Air quality measurement and management is less robust in many other parts of the world, especially in the growing cities in Africa,^15^ where emissions from diverse and complex sources are high and vary widely. The lack of routine measurement data hinders the ability of policymakers to make evidence-based policy decisions to reduce PM_2.5_ exposures and improve human health.^16^ One critical barrier is the high capital and operational costs of research-grade air pollutant monitors.^16^

Recent years have seen efforts to improve air quality monitoring in sub-Saharan Africa (SSA). There has been success in deploying low-cost sensors for short- and long-term deployments.^17^ Pope et al. (2018) conducted a 2-month measurement of PM_2.5_ and PM_10_ (coarse particulate matter) in Nairobi, Kenya, and reported exceedance of World Health Organization limits for the pollutants.^18^ Subramanian et al. (2020) investigated the effectiveness of the car-free Sunday policy in Kigali, Rwanda, by measuring PM_2.5_, carbon monoxide, and ozone with low-cost sensors, supplemented by reference BC measurements.^19^ Okure et al. (2022) used low-cost sensors to measure PM_2.5_ in Kampala.^20^ Raheja et al. (2023) compared multiple PM_2.5_ sensors over two years in Accra, Ghana.^21^ In all of these cases, the daily mean PM_2.5_ measurements in urban environments were significantly higher (23–107 μg/m^3^) than typical concentrations in the Global North.

While existing low-cost sensors can provide information on pollutant exposure, the measurements lack information about particulate matter (PM) composition or sources.^22^ PM compositional information and emission factors are critical to understanding PM sources and to designing policies that reduce PM_2.5_ exposures.^23^ In this article, we present low-cost measurements of BC at U.S. Embassy sites in SSA cities.

The U.S. Department of State collects air quality data at selected U.S. Embassies around the world to inform U.S. personnel and citizens of air quality in those regions. Many of these embassies use beta attenuation monitors (BAMs) to measure hourly ambient PM_2.5_ concentrations.^24^ BAMs collect PM_2.5_ onto a glass-fiber filter tape and estimate particle concentrations by measuring the absorption of beta radiation across samples using the Beer–Lambert law. We recently developed a nondestructive image reflectance-based method to quantify BC concentrations from BAM tape spots.^25^ In this work, we applied this method on used BAM tapes retrieved from multiple U.S. Embassies to quantify hourly concentrations of BC in multiple cities in SSA.

## 2 Materials and Methods

### 2.1 BAM Sampling and Locations

We obtained BAM tapes from U.S. Embassies in Abidjan (Côte d’Ivoire), Accra (Ghana), and two locations in Addis Ababa (Ethiopia) – Central and Jacros. The sampling period for these locations ranged between July 3 and August 29, 2020 for Abidjan, January 2 and March 2, 2023 for Accra, December 1, 2020 and January 30, 2021 for Addis Ababa Central – Summer, June 23 and August 22, 2020 for Addis Ababa Central – Winter, and August 2 and 25, 2023 for Addis Ababa Jacros – Winter. The sampling periods and locations are listed in Table S2. The locations of these embassies, as well as other U.S. Embassies in Africa that have PM measurements, are shown in Figure S1. These U.S. Embassies represent characteristics of urban environments in growing SSA cities. Additionally, we estimated BC using BAM tapes from the Lawrenceville site [Air Quality System (AQS) ID: 42–003–0008] in Pittsburgh (Pennsylvania, U.S.A.), between September 10 and October 10, 2018 as representative BC levels for an urban location in North America. The Lawrenceville site is an urban background site with both residential and commercial use. It is located downwind of the Central Business District of Pittsburgh. Consequently, local BC emissions are dominated by mobile sources. All the site descriptions are summarized in Section S2.

A BAM reports data at hourly resolution, and each spot on a used BAM filter tape represents PM_2.5_ collected in an hour. Thus, our postanalysis enables us to quantify hourly BC concentrations and explore the diurnal trends of BC.

### 2.2 BC Estimation from BAM Tape with Image Processing

We previously published a method for determining BC concentrations from BAM tapes using red light reflectance.^25^ In this method, individual BAM filter spots are photographed on top of a custom-designed reference card (Figure S2) under uniform diffused lighting using a cellphone camera (OnePlus 6, OnePlus Technology Co., Ltd., China). We applied a tailored image processing algorithm to the photo to extract the red scale value for each filter. The image processing algorithm performs geometric corrections to rectify errors from distortion, translation, or rotation errors and color corrections to account for variations in lighting conditions in the photocapturing environment. Then, we transform the red scale value to the corresponding BC area concentration (μg/cm^2^) with a precalibrated model^25^ and convert the area concentration to airborne BC concentration (μg/m^3^) using the spot area (cm^2^) and BAM flow rate (1 m^3^/h).

Each spot on a BAM filter tape corresponds to a specific hour of a day. We time-aligned the BAM spots based on the known removal date of the filter tape, and the procedure is described in Section S4. We downloaded the corresponding hourly PM_2.5_ measurements from AirNow website^26^ for the SSA cities and from the AQS website^27^ for the North American site (Pittsburgh).

## 3 Results and Discussion

### 3.1 Validation of BC Measurements with Reference Monitors

Our previous work shows a strong agreement between our BC measurements in Pittsburgh with 24 h averaged elemental carbon (EC) measurements reported every third day by the U.S. EPA’s Chemical Speciation Network (CSN).^25^ EC is another measure of carbon soot in the air and is quantified operationally as carbonaceous aerosols measured via thermal-optical methods.^28^ The comparison indicates a good performance of our method at an urban site in North America dominated by vehicular emissions.

**Figure 1:**
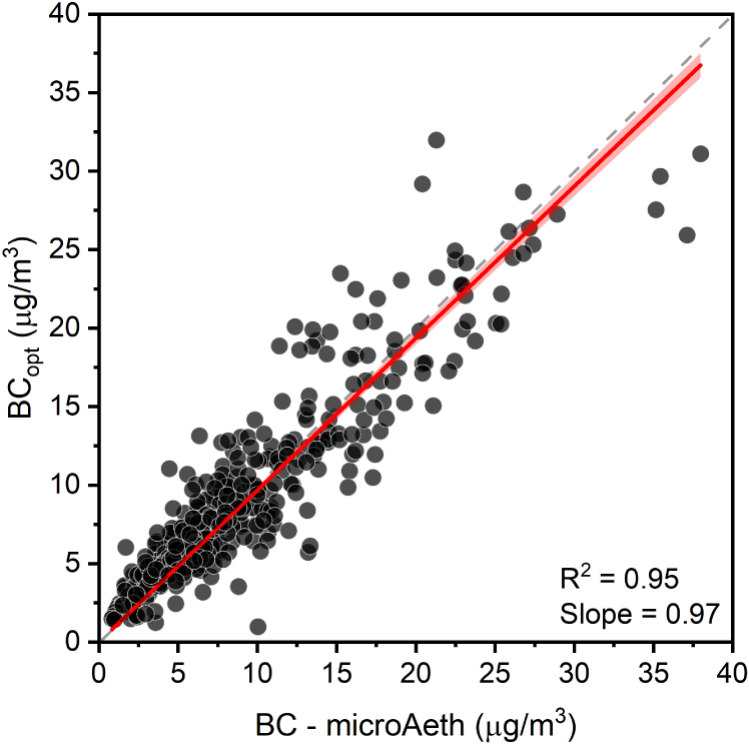
Scatter plot of hourly BC measured by our technique (BC_opt_) and by microAeth M350 (BC) at the U.S. Embassy’s warehouse at Jacros, Addis Ababa. The sampling period ranged from August 2 to 25, 2023. The dashed line represents a 1:1 line. The red line is the line of best fit for the scatter plot, and the shaded area represents the 95% confidence interval.

Air pollution characteristics in Africa are different from developed countries, and primary sources mainly include power plants, unregulated vehicular emissions, industrial emissions, combustion of solid fuels for cooking and heating purposes, and open burning of crop residues.^29^ Therefore, we performed another validation test at Addis Ababa to assess the robustness of the method at the SSA sites. NASA’s Jet Propulsion Laboratory operates a microAeth aethalometer (MA350, AethLabs, USA) next to the BAMs at each of the U.S. Embassy sites in Addis Ababa in preparation for the upcoming Multi-Angle Imager for Aerosols (MAIA) satellite mission.^30^ The MA350 measures particle absorption in 5 wavelengths (375–880 nm) at 1 min resolution. Figure 1 shows a scatter plot comparison between hourly BC estimated from the BAM tapes (BC_opt_) and BC measured by microAeth at 880 nm at Jacros between August 2 and 25, 2023.

The plot indicates a strong correlation (*R*^2^ ∼0.95) for BC hourly measurements from BAM tape with the microAeth with only a slight underestimation (∼3%) by our method. The comparison shows a root-mean-square error (RMSE), mean absolute error (MAE), and mean bias error (MBE) of 2.41 μg/m^3^, 1.70 μg/m^3^, and 0.24 μg/m^3^, respectively. The strong agreement further builds confidence in the application of our method in high BC environments, such as locations in the Global South.

### 3.2 BC and BC:PM_2.5_ Measurements

**Figure 2:**
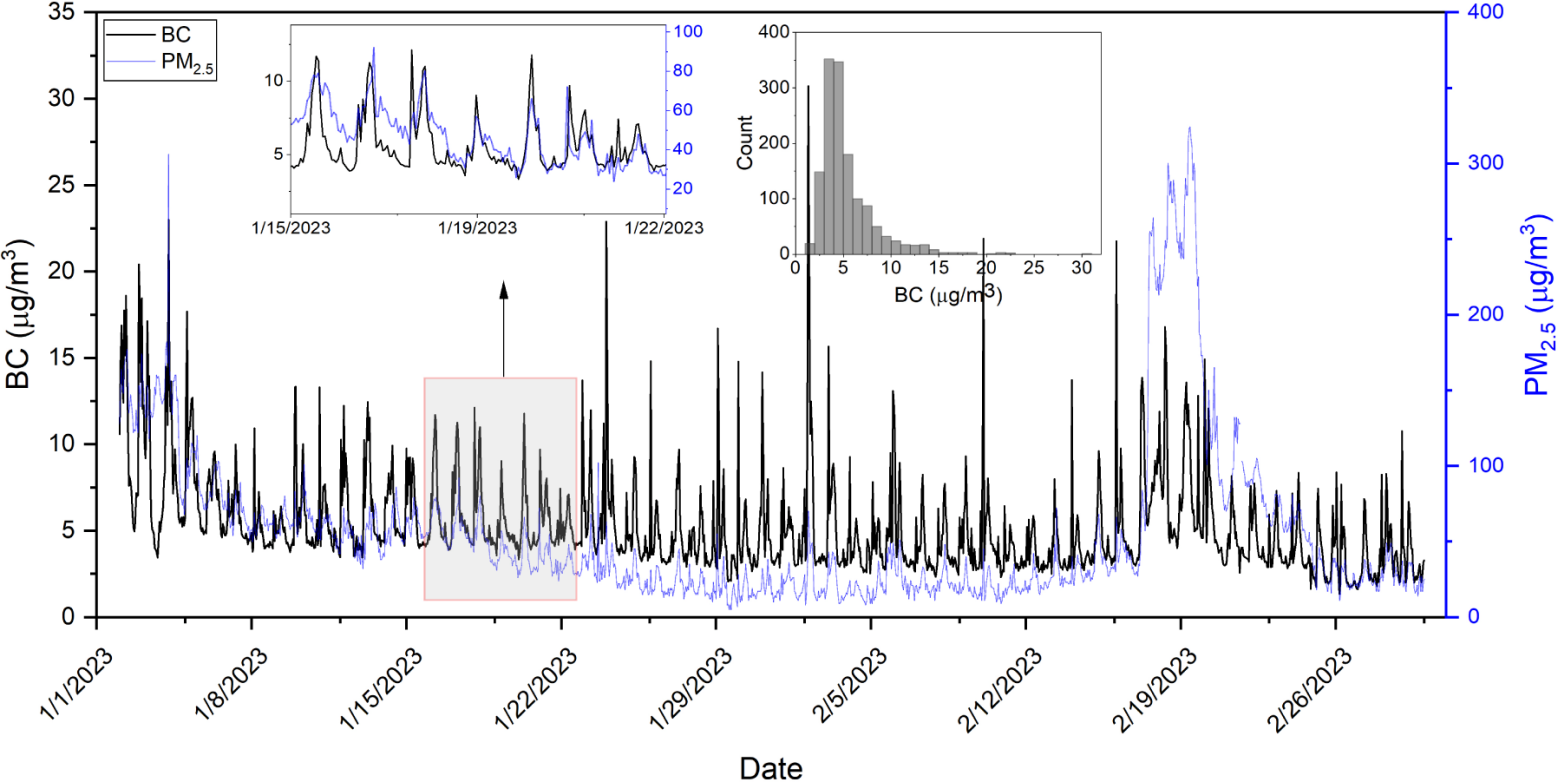
Hourly BC and PM_2.5_ time series for Accra between January 2 and March 2, 2023. The left inset plot reflects a week’s data in the time frame. The other inset plot shows the histogram of BC concentrations for the entire period.

Figure 2 shows the time series of BC and PM_2.5_ for Accra measured from January 2 to March 2, 2023. The time series for all other sites are shown in Figures S4–S7. In Figure 2, there is a clear daily trend in the levels of BC and PM_2.5_. In general, BC and PM_2.5_ are correlated. The trend and correlation are prominently visible in the time series with the inset showing a week’s measurements. Some of this correlation is from local sources like traffic, as there is a morning rush hour peak in both BC and PM_2.5_.^31,32^ Overall, *R*^2^ between hourly BC and PM_2.5_ is 0.21 (Figure S3), and BC makes up ∼14% of PM_2.5_ mass in Accra (Table S2).

There are also periods of poor correlation between BC and PM_2.5_. For example, there was a high PM event between February 17 and 21 when the mean PM_2.5_ rose to 175.1 μg/m^3^ (mean during all other times: 44.5 μg/m^3^). These high PM_2.5_ levels are likely due to a dust event as the measurements in Accra coincide with the Harmattan winds period.^31,32^ Harmattan winds are arid northeasterly trade winds that can carry large amounts of Saharan dust over West Africa and can contribute to high PM_2.5_ between November and mid-March.^33^ There was less impact on BC during this period as the mean BC during the high PM event (6.3 μg/m^3^) was only slightly higher than the mean BC at all other times (5.2 μg/m^3^). This strongly suggests a lack of dust interference in our image-reflectance method, further illustrating the robustness of our method. During these few high PM days, the *R*^2^ between PM_2.5_ and BC was 0.23 and BC was only ∼4% of PM_2.5_ whereas *R*^2^ was 0.34 and BC contributed ∼15% to PM_2.5_ mass outside of this event. The box plots for the two periods are illustrated in Figure S13, and the metrics are summarized in Table S3. This difference between the high PM_2.5_ period and all other times can also be seen in the BC:PM_2.5_ time series plot for Accra in Figure S9. The BC:PM_2.5_ ratio significantly drops during the high PM period.

**Figure 3:**
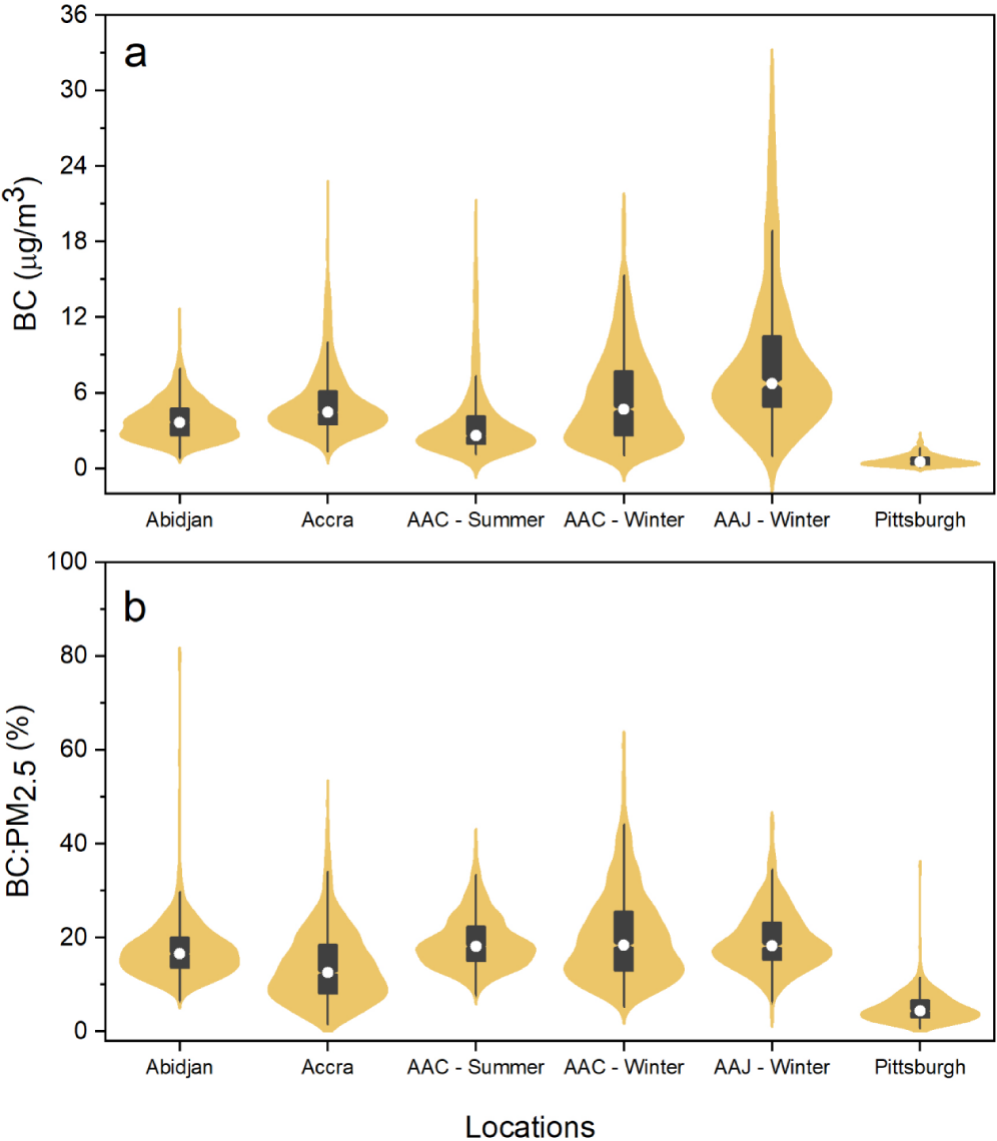
Violin plots for (a) BC and (b) BC:PM_2.5_ at Abidjan, Accra, Addis Ababa Central in the winter, Addis Ababa Central in the summer, Addis Ababa Jacros, and Pittsburgh. The violin plot envelops a box. The height of the box represents IQR, while the whiskers extend to 1.5 times the IQR at both ends. The white dot indicates the mean. The box plot is enveloped with a kernel density plot representing the distribution of the data.

Figure 3 (violin plots) and Table S2 summarize the pollutant concentrations for all sites included in this study. Average BC concentrations were significantly higher (factor of ∼7–18) and more variable in SSA cities than in Pittsburgh. Cities in SSA also showed much higher variation [higher interquartile range (IQR)] in BC, PM_2.5_, and BC:PM_2.5_ compared to Pittsburgh. BC generally made up a larger fraction of PM_2.5_ mass in SSA (∼14–20%) than that in Pittsburgh (5.6%). Winter measurements at the Addis Ababa Jacros site had the highest BC among all SSA sites (mean BC = 9.1 μg/m^3^) whereas the Addis Ababa Central site exhibited the largest BC:PM_2.5_ ratio of 20.2%. In Addis Ababa, the lowest temperatures of the year overlap with the rainy (wet) season between June and September, whereas the dry season includes between October and January.^34^ We refer to the rainy season as “winter” and the dry season as “summer” for the purpose of this study, based on the temperatures in this period. Interestingly, the summer BC measurements at Addis Ababa were among the lowest BC levels (along with Abidjan), although the BC:PM_2.5_ remained consistently high across all sites and seasons in Addis Ababa (19.2–20.2%) with marginally higher values in winter. Biomass, including charcoal, provides more than 80% of the household energy in Addis Ababa.^35^ Therefore, these high BC levels in winter are potentially caused by a significant increase in the use of solid biomass fuels for space heating, coupled with temperature inversions and reduced atmospheric circulation during winter in Addis Ababa.^34,36^ Among SSA sites, the BC:PM_2.5_ level was the lowest in Accra (∼14%) though it was still 2.5 times that in Pittsburgh (∼5.6%).

The data in Abidjan cover the period of July–August 2020. The *R*^2^ between BC and PM_2.5_ in Abidjan was ∼0.55. The mean BC concentration was 3.85 μg/m^3^, and BC made up 17.5% of the PM_2.5_ mass. The high BC and high correlation between BC and PM_2.5_ suggest that this site is influenced by traffic emissions.^37^ A study by Gnamien et al. (2023) measured similar EC levels of 3.2 ± 1.7 μg/m^3^ and EC composed 18.2% of PM_2.5_ in Abidjan.^38^ Kouassi et al. (2021) reported that daily BC at Abidjan was 5.31 ± 2.5 μg/m^3^ in 2018.^39^

Abidjan experiences four major seasons in a year, namely the great dry season (December–March), the great rainy season (April–July), the small dry season, (August–September), and the small rainy season (October–November).^37^ Our measurements include one month each for rainy/wet (July) and dry (August) seasons. We observed that BC, PM_2.5_, and BC:PM_2.5_ measurements were systematically higher in the wet season (mean levels of 4.2 μg/m^3^, 22.6 μg/m^3^, and 19.2%, respectively) than in the dry season (mean levels of 3.4 μg/m^3^, 21.5 μg/m^3^, and 15.9%, respectively) at Abidjan. The box plot comparing BC, PM_2.5_, and BC:PM_2.5_ measurements and the corresponding metrics are available in Section S9 The mean wind speed (18.2 and 16.9 km/h) and mean rainfall (net rainfall ∼1.2–5.4 mm) in both seasons were similar. The lower BC and BC:PM_2.5_ levels in the dry season could be from reduced traffic activities resulting from summer holidays in schools between late July and August.^39^

Our measurement period at Addis Ababa consists of a winter season (June 23 to August 22, 2020) and a summer season (December 1, 2020 to January 30, 2021). Addis Ababa is an interesting site with both ends of extreme BC measurements. In this study, Addis Ababa allows a seasonal comparison of pollutant concentrations in an urban environment in Africa. Winter BC levels at Addis Ababa Jacros showed the highest BC levels (mean ∼9.1 μg/m^3^); however, average BC concentrations in summer at Addis Ababa Central were the lowest (∼3.9 μg/m^3^) across all SSA cities. Seasonal comparison at Addis Ababa Central shows a 1.5 times higher mean BC and 1.4 times higher mean PM_2.5_ concentration in the winter than in the summer. Addis Ababa in the winter showed the highest fraction of BC:PM_2.5_ (∼20%) across SSA sites as well. Despite the large variations in pollutant levels, the average BC:PM_2.5_ for the two seasons remained nearly constant across all Addis Ababa sites and seasons (<5% change).

Both BC and BC:PM_2.5_ levels in Addis Ababa show significantly higher variability (higher IQR) in the winter than in the summer. This high variation could be caused by rapid fluctuation in the boundary layer height throughout the day in the winter as opposed to in the summertime. The highest correlation between BC and PM_2.5_ (*R*^2^ ∼0.82) was observed for measurements at the Addis Ababa Central site during the summertime, a period of lowest mean pollutant concentrations in SSA sites. Summer represents a period of higher boundary layer height with better mixing of pollutants due to atmospheric circulation. This leads to minimal interference of background pollutant levels into BC and PM_2.5_ measurements, yielding better correlation in the summertime.

### 3.3 Diurnal Pattern of BC at SSA Cities

**Figure 4:**
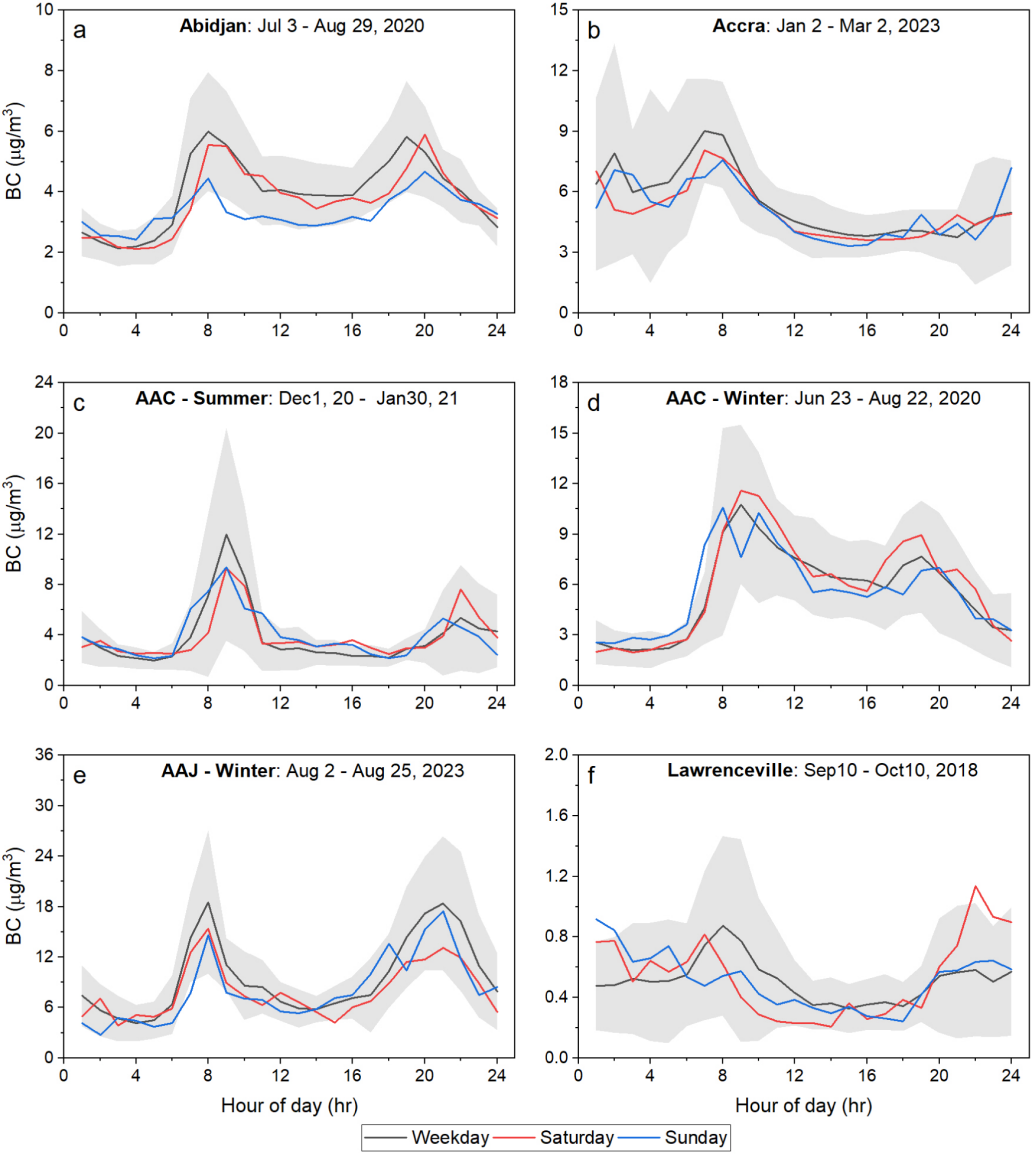
Diurnal pattern for the median BC concentrations. Separate diurnal patterns corresponding to weekday, Saturday, and Sunday are plotted for (a) Abidjan, (b) Accra, (c) Addis Ababa Central site in summer (AAC-Summer), (d) Addis Ababa Central site in winter (AAC-Winter), (e) Addis Ababa Jacros site in winter (AAJ-Winter), and (f) Lawrenceville site in Pittsburgh. The gray-shaded area shows 10th and 90th percentiles for the weekday diurnal trend.

We used hourly BC measurements to determine the diurnal trends for each SSA site. Figure 4 shows diurnal patterns for median BC concentrations for weekday, Saturday, and Sunday at each location in SSA and Pittsburgh.

All cities show an expected morning rush hour peak in BC. The morning rush hour is attributed to vehicular emissions from people commuting to work. However, the presence of an evening rush hour peak is site-dependent. Another important factor contributing to this BC trend is the diurnal variation of the boundary layer height. The lowest boundary layer height, resulting from temperature inversions, typically coincides with the morning rush hour,^40^ which causes an increase in BC concentrations.

**Figure 5:**
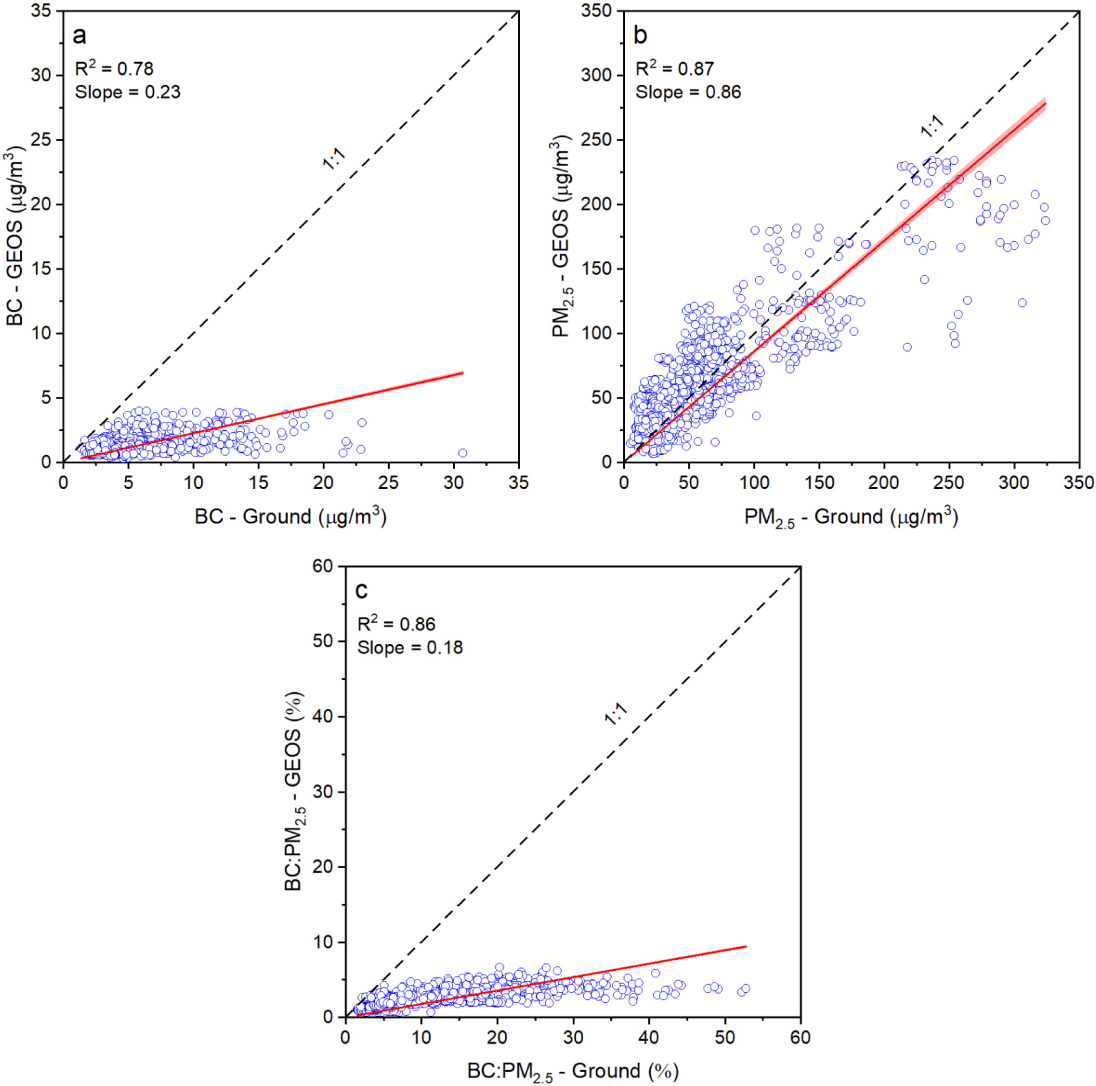
Scatter plot comparison between hourly ground monitor measurements and GEOS-CF estimates for Accra. (a) Scatter plot between measured BC (indicated as “BC-Ground”) and BC from GEOS-CF (indicated as “BC-GEOS”), (b) scatter plot for PM_2.5_ from AirNow and GEOS-CF, and (c) scatter plot comparison of BC:PM_2.5_ for ground measurements and the GEOS-CF.

We also expect a weekday–weekend difference, which majorly depends on whether Saturday and Sunday are workdays at a target site. Some of the sites show distinctly lower peak BC concentrations for one or more of the weekend days, potentially due to fewer people working and commuting on weekends. This difference is further investigated by applying a nonparametric Mann–Whitney U test (or Wilcoxon rank sum test) on the diurnal BC dataset during weekday, Saturday, and Sunday for each site. The test suggests no statistically significant difference in the overall diurnal trends of BC among different day categories for any of the sites with a 95% confidence interval (Table S5). This is likely influenced by small differences in BC concentrations during non-peak hours of a day, which represents most of a day. At Abidjan, the *p*-value of 0.056 for the Weekday–Sunday comparison is very close to the 0.05 threshold for the 5% significance level. Figure 5a suggests a noticeable difference in BC concentrations between weekdays and Sundays as well as between Saturdays and Sundays in Abidjan.

The Pittsburgh site is an example of an urban environment in North America. It is an urban residential area downwind of the Central Business District. BC concentrations are dominated mostly by traffic emissions. The morning rush hour peak is highest (median BC = 0.87 μg/m^3^) during weekdays. The rush hour peak is weaker on Saturday and especially on Sunday, where there is no discernible morning increase in BC. On both weekdays and weekends, the BC concentration drops in the afternoon and gradually rises around 7 pm due to changes in the boundary layer height. Evening and nighttime BC (7 pm to midnight) are higher on Saturdays than on Sundays and weekdays. This may be due to local commercial activities (e.g., restaurant emissions and traffic to those restaurants).

The SSA locations show much higher concentration levels compared to Pittsburgh. In Abidjan, weekday BC concentration is lowest during the night (∼2 μg/m^3^), slowly rises in the morning, and peaks at ∼6 μg/m^3^ around 8 am during the morning rush hour. A similar peak in BC, likely from traffic, can be observed around 8–9 am on Saturday, a working day in Abidjan. The Abidjan U.S. Embassy site is located in the downtown area only ∼280 m from a major traffic intersection and is likely impacted by local traffic and restaurant emissions. Thus, the evening peak (7–8 pm) is mainly caused by emissions from rush hour traffic and cooking activities in restaurants. Sunday is not a workday in Abidjan. Thus, both the morning and evening BC peaks are smaller on Sundays than on other days.

Accra has a large morning BC peak (∼9 μg/m^3^) between 7 and 8 am. The BC levels drop as the day progresses but remain near ∼4 μg/m^3^ throughout the day. Many diplomatic missions, including the U.S. Embassy in Accra, and military establishments are located in the neighborhood near the embassy. The embassy is situated ∼180 m away from a major traffic circle, sandwiched by two parallel arterial roads running at ∼150 m on each side, and sits ∼1.5 km south of the Kotoka International Airport. Thus, the site is continuously exposed to high levels of road traffic- and aviation-related emissions, which explains such high BC levels throughout a day. Interestingly, a peak was observed between midnight and 3 am on all days. This peak is possibly due to emissions from diesel generators used by the establishments during late-night or early-morning power outages. Another possibility is illicit nighttime waste burning as a medium of waste disposal by locals living in small unauthorized settlements near the arterial roads.

Morning peaks in Addis Ababa were slightly later than in the other cities and occurred between 8 and 10 am during both winter and summer. Addis Ababa shows the highest peak concentrations compared to any other cities in Africa, which can be attributed to the closest proximity of the sites to major roadways. The Central site is the main embassy location, situated ∼85 m from Algeria Steet (a major arterial road) and ∼1.6 km from a traffic circle connecting two perpendicular arterial roads. In the summer, BC concentrations at this site gradually decrease overnight to ∼2 μg/m^3^, rising sharply around 6 am to peak at 9 am and quickly dropping to the same level by noon. Both seasons have distinct evening or nighttime BC peaks. Additionally, the winter season diurnal shows elevated baseline BC concentrations. These consistently high BC concentrations could be explained by additional emissions from solid biofuel combustion to meet heating requirements in the winter season. In both seasons, Sunday shows a lower BC concentration for the entire day. The Jacros site in Addis Ababa shows the highest peak concentrations of BC (∼18 μg/m^3^), which can be attributed to its location in a traffic-centric area.

### 3.4 Comparison of the Ground BC Measurements with Goddard Earth Observing System Composition Forecast (GEOS-CF) Outputs

We compared our hourly ground measurements with the hourly outputs from the GEOS-CF model for BC, PM_2.5_, and BC:PM_2.5_ at all target sites. The hourly GEOS-CF outputs for BC and PM_2.5_ are available globally at 0.25° × 0.25° (25 × 25 km) grid resolution, estimated at 35% relative humidity. Keller et al. (2021) provide a detailed description of the model.^41^ The averaged value of a parameter from a GEOS-CF grid enclosing a target location is compared with the ground measurement at the location.

Figure 5 shows a comparison between hourly BC, PM_2.5_, and BC:PM_2.5_ for Accra. Figure 5 shows a strong correlation (*R*^2^ ∼0.87) between PM_2.5_ from GEOS-CF and ground measurements in Accra. The model underestimates PM_2.5_ by ∼14% (slope ∼0.86) with very low normalized root-mean-square error (NRMSE), normalized mean bias error (NMBE), and normalized mean absolute error (NMAE) values of 0.5, 0.05, and 0.35, respectively. The errors are normalized with the mean of ground observations for corresponding parameters. The detailed approach for calculating these errors is included in Section S11. The agreement is weaker for BC. For BC, *R*^2^ is 0.78, and GEOS-CF underpredicts BC concentrations by nearly a factor of 5 (slope ∼0.23) with higher errors (NRMSE: 0.9, NMBE: −0.73, and NMAE: 0.73) than for PM_2.5_. This underestimation in BC leads to almost a 5 times lower model-derived BC:PM_2.5_.

Overall, the model performance was worse for BC than for PM_2.5_ in the cities studied here. Table S7 summarizes the statistical metrics for BC, PM_2.5_, and BC:PM_2.5_ comparison between the ground and GEOS data as an indicator for the performance of GEOS-CF. Abidjan showed a good correlation between the ground monitor and GEOS-CF predictions with *R*^2^ for BC, PM_2.5_, and BC:PM_2.5_ of 0.72, 0.87, and 0.86, respectively. However, GEOS estimated only 56% of the hourly PM_2.5_ and 17% of the hourly BC measured by the ground monitor, the lowest across all of the sites. NMBE for BC of −0.8 indicates a systematic underestimation of BC concentrations by the model.

In Addis Ababa, NRMSE is high for BC in both seasons (winter: 1.14, summer: 1.03). The correlation between the model and the ground data for BC is slightly higher in winter (*R*^2^ = 0.34) than in summer (*R*^2^ = 0.42). The NMBE for BC (winter: −0.65, summer: −0.69) is much higher than that for PM_2.5_ (winter: −0.08, summer: 0.01) indicating that the model underestimates BC.

The Pittsburgh site shows the highest NRMSE (∼1.15) and NMAE (0.87) for BC across all the sites. The NMBE for BC (0.67) at this site reveals an overestimation, contrasting with the underestimation observed in the other locations. The mean BC concentration at the Pittsburgh site is significantly lower (<1 μg/m^3^) than the SSA sites which can potentially introduce greater estimation uncertainty due to higher relative error at smaller concentration levels.

Overall, the GEOS-CF model underestimates BC in the SSA cities. NMBE ranges from −0.65 to −0.8 for the SSA locations, indicating a large underestimation for hourly BC. The lower estimates by the model in SSA sites can be partially explained by spatial resolution. The model predicts averaged concentrations for a 25 × 25 km grid, whereas the ground measurements are point measurements. Nevertheless, grids for all target locations encompass urban environments, and the U.S. Embassies are typically at urban background locations. Thus, the point measurements at these locations could represent grid-averaged concentrations. The persistent negative bias in the model predictions may therefore indicate that the model underestimates the local BC emissions for the SSA cities. Continuous ground-based BC measurements at these locations, especially for BC, might prove helpful in mitigating these differences between the model and ground measurements.

## 4 Implications

This study presents a method to measure hourly BC concentrations by leveraging already existing BAMs (PM_2.5_ monitors) at U.S. Embassies. We applied image-processing techniques to PM spots on previously used BAM tapes to estimate ambient BC concentrations and validated this technique with a reference method at the U.S. Embassy warehouse in Addis Ababa. Hourly BC in SSA cities was much higher (mean BC: 3.9–9.1 μg/m^3^, mean BC:PM_2.5_: 14–20%) compared to that in Pittsburgh (mean BC: 0.5 μg/m^3^, mean BC:PM_2.5_: 5.6%).

These measurements are crucial to understanding the severity of air pollution in developing countries and underscore the need for more ground monitors to assist in policymaking. The BC data, along with PM_2.5_ measurements by BAMs, allow us to quantify the contribution of combustion-based emissions (BC:PM_2.5_) in an environment. BC:PM_2.5_ levels in SSA cities (mean BC:PM_2.5_: 20%) were as high as 4 times those in Pittsburgh (mean BC:PM_2.5_: 5.6%). Our BC measurements provide insight into seasonal (wet–dry or summer–winter) differences in BC levels and their effects on BC:PM_2.5_. We also use the measurements at the Accra site to investigate the effect of Harmattan winds on BC and BC:PM_2.5_.

The hourly data also allow us to determine diurnal trends for BC at all locations in this study. These trends allow us to effectively monitor combustion emissions throughout the day for all days of the week. For instance, the diurnal BC pattern for Accra indicated a unique BC peak early in the morning, consistently occurring throughout all days of the week, which could help in identifying and mitigating these isolated sources.

Satellite data and chemical transport models have been used to estimate PM_2.5_ and its composition to assess health impacts in SSA regions.^42^ Ground-level data are critical to calibrate the model performance.^43,44^ However, model efficacy is uncertain in many parts of Africa because of a lack of ground data for evaluating its performance and there is a need for more ground-level composition data to evaluate the performance of these models.^42,43,45−49^ Adding ground-level PM and speciation measurements could further help validate model outputs and evaluate chemical transport models for improved outputs.

This BC estimation technique proved to be effective in acquiring useful BC data in developing nations to mitigate the lack of comprehensive air quality data, which is an obstacle to effective policymaking and intervention strategies. Key advantages of our technique include its low cost and scalability. In particular, the existing monitoring network operated by the U.S. State Department to measure ambient PM_2.5_ using BAMs at several U.S. Embassies worldwide, especially in LMICs, presents a prime opportunity for this application. This cellphone-based BC quantification requires a phone or webcam and a reference card. The State Department could collaborate with research groups in local universities to measure BC with this technique by utilizing the used BAM filter tapes, which would otherwise be discarded. Thus, this method can be conveniently applied at nearly zero cost to both long-term samples such as the embassy BAMs presented here and filter samples collected by regulatory agencies or community groups. Implementing this approach could unveil the levels of BC, among the most toxic components of PM_2.5_, in several under-monitored regions of the world, as demonstrated in this study.

## Supporting Information Available

Map of Africa showing all the cities in SSA where U.S. Embassies measure PM_2.5_ (Figure S1); description of sampling sites (Section S2) and their geographical information (Table S1); description of the reference card used in this study (Section S3); process of assigning date and time for BC from BAM spots (Section S4); scatter plots between BC and PM_2.5_ (Section S5) and pollutant sampling summary for the cities in this study (Table S2); BC and PM_2.5_ time series for the measurement sites (Figures S4–S7 in Section S6); BC:PM_2.5_ time series for the measurement sites (Figures S8–S12 in Section S7); box plot comparison of hourly BC, PM_2.5_, and BC:PM_2.5_ levels in “low” and “high” PM events at Accra (Figure S13, Section S8); mean hourly BC, PM_2.5_, and BC:PM_2.5_ for the “low” and “high” dust events in Accra (Table S3, Section S8); box plot comparison of hourly BC, PM_2.5_, and BC:PM_2.5_ levels in wet and dry seasons at Abidjan (Figure S14, Section S9); mean weather and pollution parameters and for wet and dry seasons in Abidjan (Table S4, Section S9); analyzing variations in diurnal trend across different days of the week (Section S10); Mann–Whitney U test summary to identify significant difference in diurnal trend of BC between weekday, Saturday, and Sunday (Table S5); error metrics description to compare measured and GEOS-CF BC (Section S11); site locations, their coordinates, and coordinates for the midpoint of the corresponding GEOS-CF grid cell (Table S6, Section S12); statistical metrics to compare hourly ground measurements with GEOS-CF estimates BC, PM_2.5_, and BC:PM_2.5_ (Table S7, Section S12) (PDF)
