# Supplementary material for: Low-Cost Hourly Ambient Black Carbon Measurements at Multiple Cities in Africa
Source: Environ Sci Technol. 2024 Jul 2;58(28):12575–84. doi: 10.1021/acs.est.4c02297 (PMC11256757; doi:10.1021/acs.est.4c02297)
Supplement: Supplementary file 1 — es4c02297_si_001.pdf [file es4c02297_si_001.pdf]

Supplementary Information

Low-Cost Hourly Ambient Black Carbon Measurements at Multiple Cities in Africa

Abhishek Anand[1,2], N'Datchoh Evelyne Touré[3], Julien Bahino[3], Sylvain Gnamien[3], Allison Felix Hughes[4], Raphael E Arku[5], Victoria Owusu Tawiah[6], Araya Asfaw[7], Tesfaye Mamo[7], Sina Hasheminassab[8], Solomon Bililign[9], Vaios Moschos[9], Daniel M. Westervelt,[10] Albert A. Presto[1,2]*

[1]Center for Atmospheric Particle Studies, Carnegie Mellon University, Pittsburgh, PA, USA

[2]Department of Mechanical Engineering, Carnegie Mellon University, Pittsburgh, PA, USA

[3]Université Félix Houphouët-Boigny, Abidjan, Côte d'Ivoire

[4]University of Ghana, Accra, Ghana

[5]Department of Environmental Health Sciences, University of Massachusetts Amherst, Amherst, MA, USA

[6]Department of Meteorology & Climate Science, Kwame Nkrumah University of Science and Technology, Kumasi, Ghana

[7]Institute of Geophysics, Space Science and Astronomy, Addis Ababa University, Addis Ababa, Ethiopia - P.O. Box: 1176

[8]Jet Propulsion Laboratory, California Institute of Technology, Pasadena, CA, USA

[9]Department of Physics, North Carolina A&T State University, Greensboro, NC, USA

[10]Lamont Doherty Earth Observatory, Columbia University, New York, NY, USA

*Corresponding author: apresto@andrew.cmu.edu

Summary of the Supporting Information

Number of pages: 24

Figures: S1 – S14

Tables: S1 – S6

Table of Contents

S1. Sampling locations 3

S2. Site description 4

S3. Reference Card 6

S4. Assigning date and time for BC from BAM spots 7

S5. Scatter plots and measurements summary for BC and $PM_{2.5}$ for all target sites 8

S6. BC and $PM_{2.5}$ timeseries at the measurement sites 10

S7. BC:$PM_{2.5}$ timeseries at the measurement sites 14

S8. Low and high PM event comparison of BC, $PM_{2.5}$ and BC:$PM_{2.5}$ for Accra 19

S9. Seasonal comparison of BC, $PM_{2.5}$ and BC:$PM_{2.5}$ for Abidjan 20

S10. Metrics description 21

S11. Extraction of GEOS-CF data for the sites 26

REFERENCES 27

S1. Sampling locations

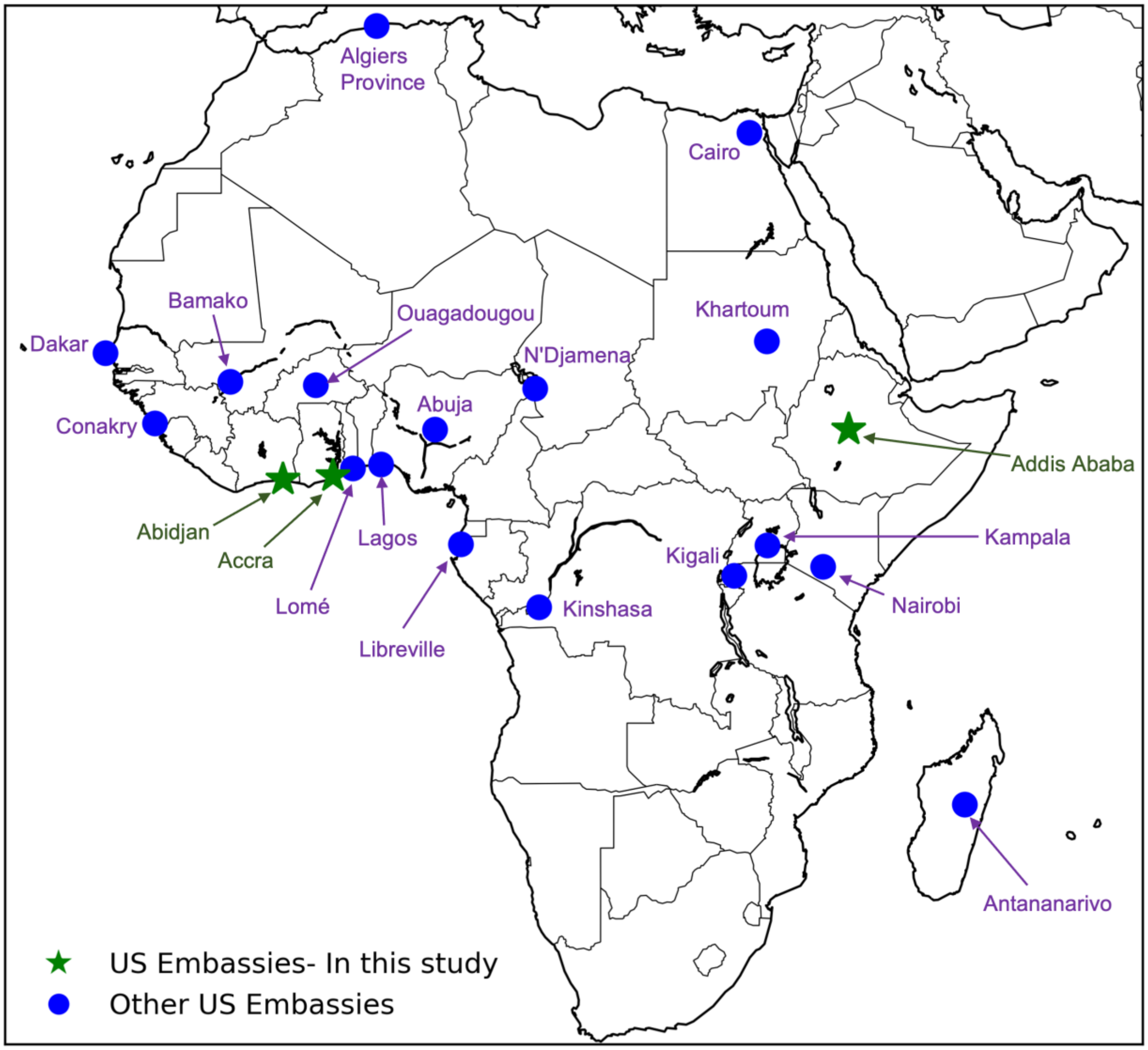

Figure S1: The map shows all the cities in SSA where U.S. Embassies measure $PM_{2.5}$. The green stars indicate the target SSA locations in this study and the blue circles are remaining sites in SSA with $PM_{2.5}$ measurements.

S2. Site description

Table S1: Sampling sites and their geographical information

Site location	Coordinates	Notes
Abidjan, Côte d'Ivoire (ABJ)	5.335040, -3.976045	U.S. Embassy
Accra, Ghana (ACC)	5.579480, -0.170623	U.S. Embassy
Addis Ababa Central, Ethiopia (AAC)	9.058586, 38.760151	U.S. Embassy
Addis Ababa Jacros, Ethiopia (AAJ)	9.011387, 38.820928	U.S. Embassy warehouse
Lawrenceville Site, Pittsburgh PA, USA (PIT)	40.465420, -79.960757	A U.S. EPA Chemical Speciation Network (CSN) site. It is also a monitoring site for the Allegheny County Health Department.

The $PM_{2.5}$ data were extracted from the Beta Attenuation Monitors (BAMs) and BC was estimated from the BAM filter tapes.. The BAMs are installed at U.S. Embassies at sites in SSA. The BAM site at Pittsburgh is one of the Chemical Speciation Network (CSN) site. The geographic information on these sites is briefly summarized in Table S1 and further detailed below.

Abidjan, Côte d'Ivoire

Côte d'Ivoire is located in the Western Africa. U.S. Embassy at Abidjan is positioned ~250 m from a major traffic circle, ~500 m north of the Ébrié Lagoon, and only 8.9 km north of the Gulf of

Guinea. It is located within the city's prominent urban area, where emissions are primarily influenced by vehicular traffic. The city experiences high dust episodes from occasional Harmattan winds carrying Saharan dust between November to March.

Accra, Ghana

Accra is another location in Western Africa among our measurement sites. The U.S. Embassy is in the Cantonment suburb, situated ~180 m away from a major traffic circle and sandwiched by two parallel arterial roads running at ~150 m on each side. The Embassy is ~1.5 km south of the Kotoka International Airport, and ~3.25 km north of the Gulf of Guinea. The city experiences high dust episodes from occasional Harmattan winds carrying Saharan dust between November to March.

Addis Ababa Central, Ethiopia

Ethiopia is an East African country. The U.S. Embassy is only 85 m from, Algeria St, a major arterial road and ~1.6 km from a traffic circle connecting arterial roads.

Addis Ababa Jacros, Ethiopia

The BAM in the Jacros area of Addis Ababa is hosted by the warehouse facility of the U.S. Embassy at Addis Ababa and is located merely at 50 m away from a major arterial road and ~800 m from a south of the A2 highway.

Pittsburgh, USA

The BAM is located at the Lawrenceville Allegheny County Health Department monitoring station and hosts $PM_{2.5}$ speciation as a part of the Chemical Speciation Network (CSN) initiative by the U.S. EPA. The station is located in an urban residential area downwind of Central Business District. The air pollution at the station is dominated by vehicular emissions.

S3. Reference Card

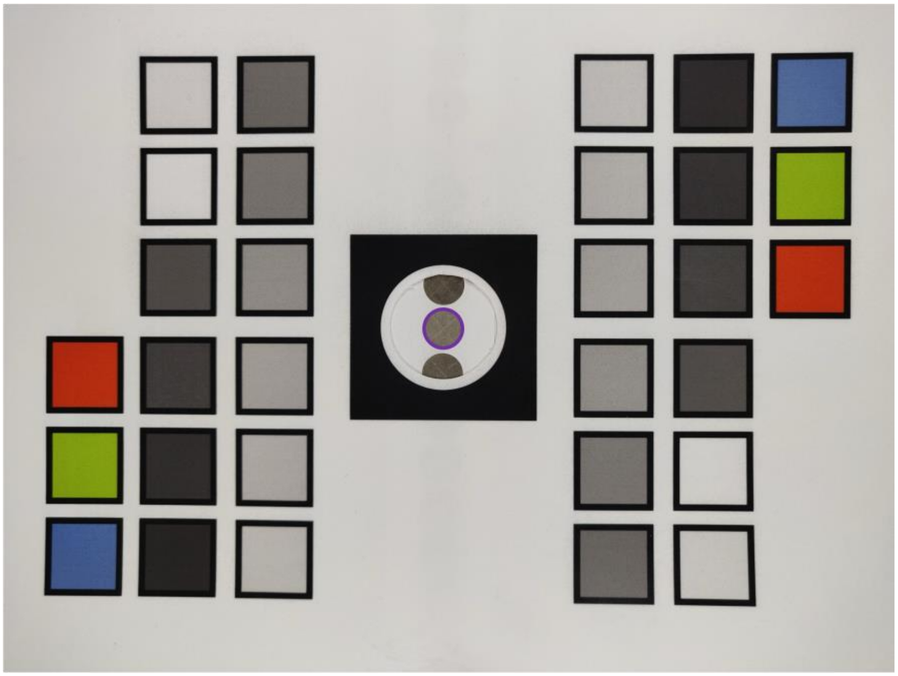

Figure S2: BAM tape spots are aligned against the reference card (shown above) one at a time and the assembly is then photographed. Particulate deposit spots are placed at the center circle. The purple circle in the center shows the target area for analysis. Red, green and blue (RGB) color channels are shown for all the square boxes. The 3 colored boxes in the reference card are used for color calibration, whereas the Red channel of each grayscale box (R values for the gray boxes are 75, 91, 107, 123, 139, 155, 171, 187, 203, 219, 235, 251) corresponds to a unique BC concentration.

S4. Assigning date and time for BC from BAM spots

A BAM collects particles on a filter tape at a fixed flow rate every hour and measures $PM_{2.5}$ by quantifying attenuation through the particle deposits. While there are other versions of BAMs available, we analyzed tapes from a BAM 1020 (Met One Instruments Inc.) used widely by the U.S. State Department to measure $PM_{2.5}$ concentrations at U.S. Embassies and Consulates. Therefore, BAM refers to BAM 1020 from Met One Instruments in our measurements.

The U.S. Embassy stores BAM tapes in their original boxes after use and labels each box with the date the corresponding was removed after use. Sometimes, there are torn parts of tapes in the boxes which makes it difficult to narrow down the exact date and time for BAM spots. Spots on BAM filter rolls exist as set of 24 equally spaced spots representing 24 hours of a day. Each set of 24 spots are separated from the next 24 spots by a relatively larger space. We discarded the broken tapes (accounting total number of spots, hence hours of BC data, discarded) and started the analysis for the first continuous 24 spots from the tape end. Additionally, we excluded the days with fewer than 24 spots during analysis to avoid estimation errors from sampling biases caused by unscheduled interruptions of BAM operations as well as erroneous date and time assignment for the spots.

We start by assuming the removal date as the last date for the measured BAM spots and correspondingly assign date and time for each BC estimation during the sampling period. Then, we download hourly $PM_{2.5}$ measurements from AirNow website[1] for the SSA sites and from the Air Quality System (AQS) website[2] for the Lawrenceville site in Pittsburgh. We chose the coefficient of determination (R^2) between the hourly $PM_{2.5}$ from AirNow and BC_{opt} as the indicator, and recorded R^2 by moving up the last datetime for the measurement period. The date-time assignment with the highest R^2 value for that measurement period was finalized.

S5. Scatter plots and measurements summary for BC and $PM_{2.5}$ for all target sites

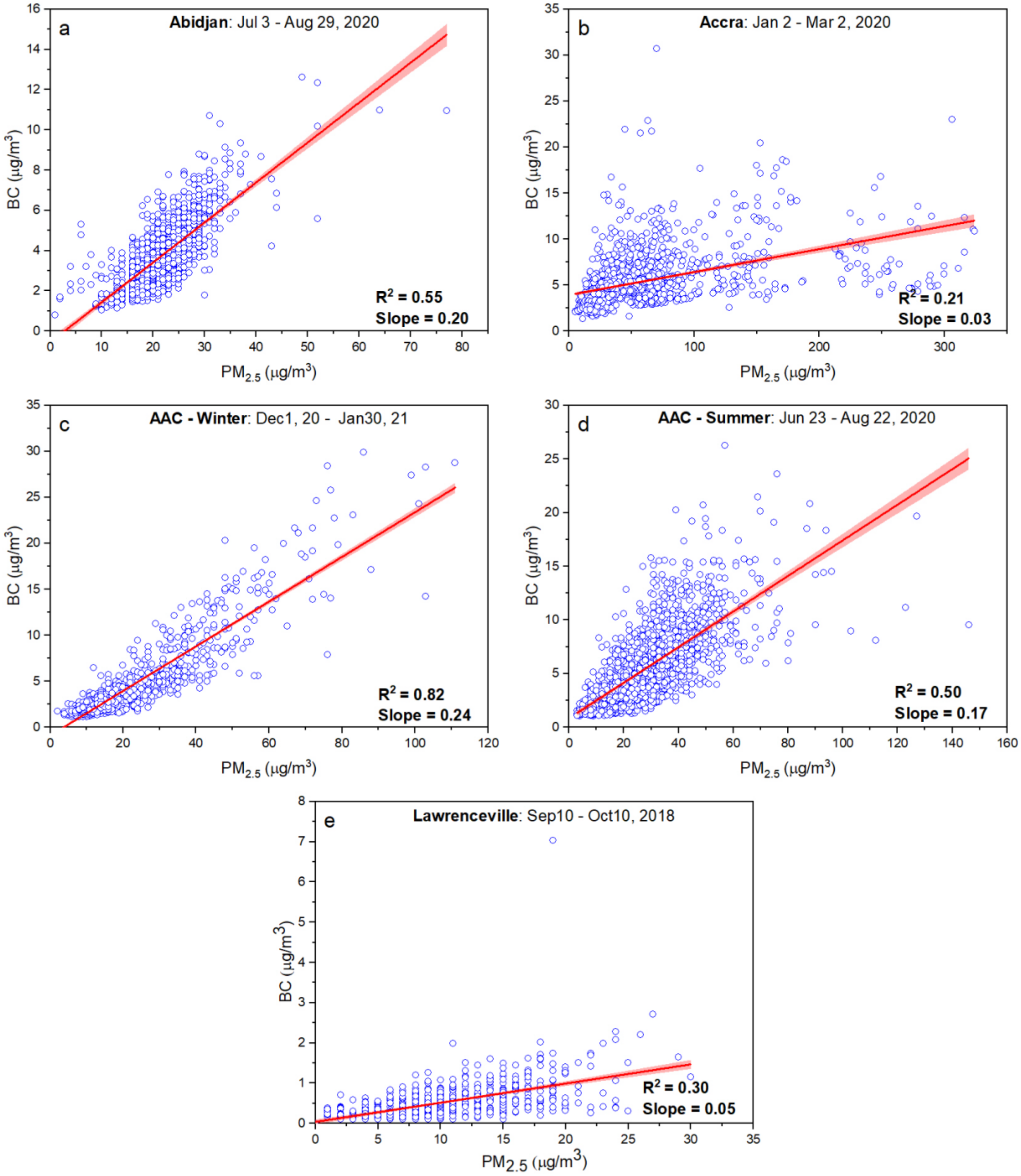

Figure S3: Scatter plot between hourly BC estimated with BAM tapes and hourly BAM-based $PM_{2.5}$ concentrations retrieved from AirNow. The red line is the best linear fit for the scatter plot, enveloped by the 95% confidence band for the fit.

Table S2: Sampling summary for the target cities in SSA. N indicates the number of sampling days. μ_{BC}, $\mu_{PM2.5}$ and μ_{ratio} represents mean hourly BC, $PM_{2.5}$ and BC:$PM_{2.5}$, respectively. $R^2_{particles}$ values indicate correlation between hourly BC and $PM_{2.5}$ for a location during the sampling duration.

**Locations**	**Sampling period**	**N, days (N_{Sat}, N_{Sun})**	**$R^2_{particles}$**	**μ_{BC} (μg/m^3)**	**$\mu_{PM2.5}$ (μg/m^3)**	**$\mu_{BC:PM2.5}$ (%)**
Abidjan, Cote D'Iviore	Jul 3, 2020 – Aug 29, 2020	58 (11, 10)	0.55	3.85	22.0	17.5
Accra, Ghana	Jan 2, 2023 – Mar 2, 2023	59 (8, 8)	0.21	5.33	38.1	13.9
Addis Ababa Central site, Ethiopia - Winter (AAC-W)	Jun 23, 2020 – Aug 22, 2020	60 (8, 8)	0.5	5.63	27.9	20.2
Addis Ababa Central site, Ethiopia - Summer (AAC-S)	Dec 1, 2020 – Jan 30, 2021	60 (8, 8)	0.82	3.89	20.3	19.2
Addis Ababa Jacros site, Ethiopia – Winter (AAJ-W)	Aug 2 – 25, 2023	24 (3, 3)	0.76	9.14	42.4	19.7
Lawrenceville, Pittsburgh, PA, USA	Sep 10, 2018 – Oct 10, 2018	31 (4, 4)	0.30	0.52	10.29	5.6

S6. BC and $PM_{2.5}$ timeseries at the measurement sites

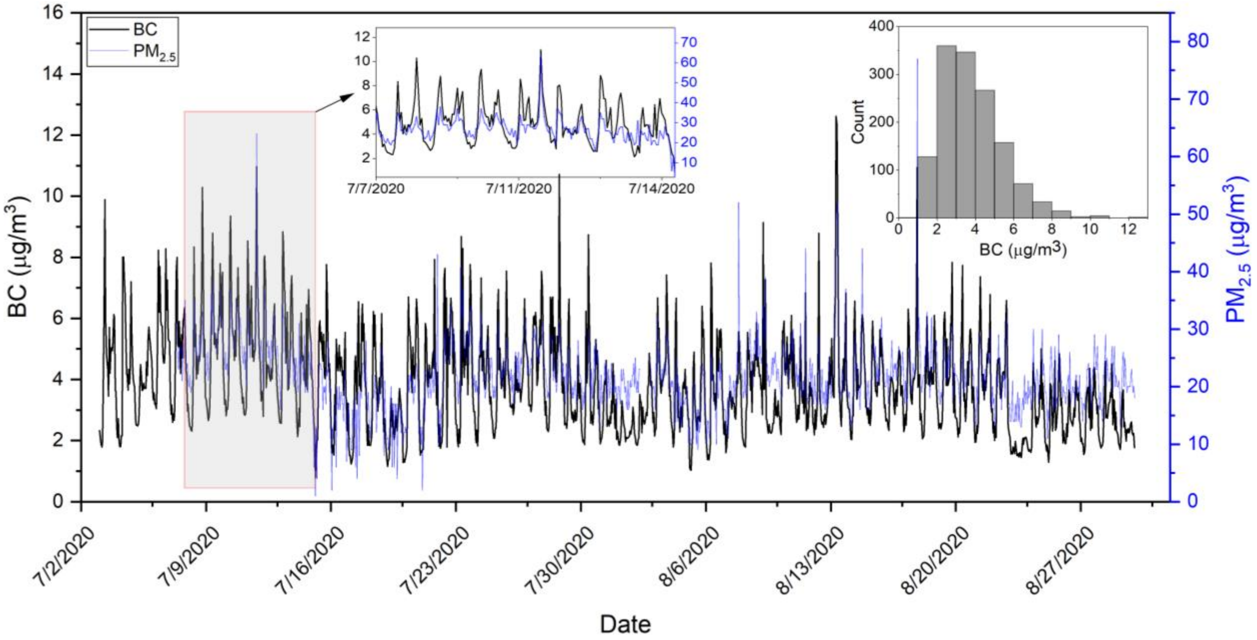

Figure S4: Hourly BC and $PM_{2.5}$ timeseries for Abidjan between July 3 – August 29, 2020. The timeseries inset plot shows a week's data in the shaded gray box. The histogram inset plot shows the distribution of BC concentrations for the Abidjan site during the sampling period.

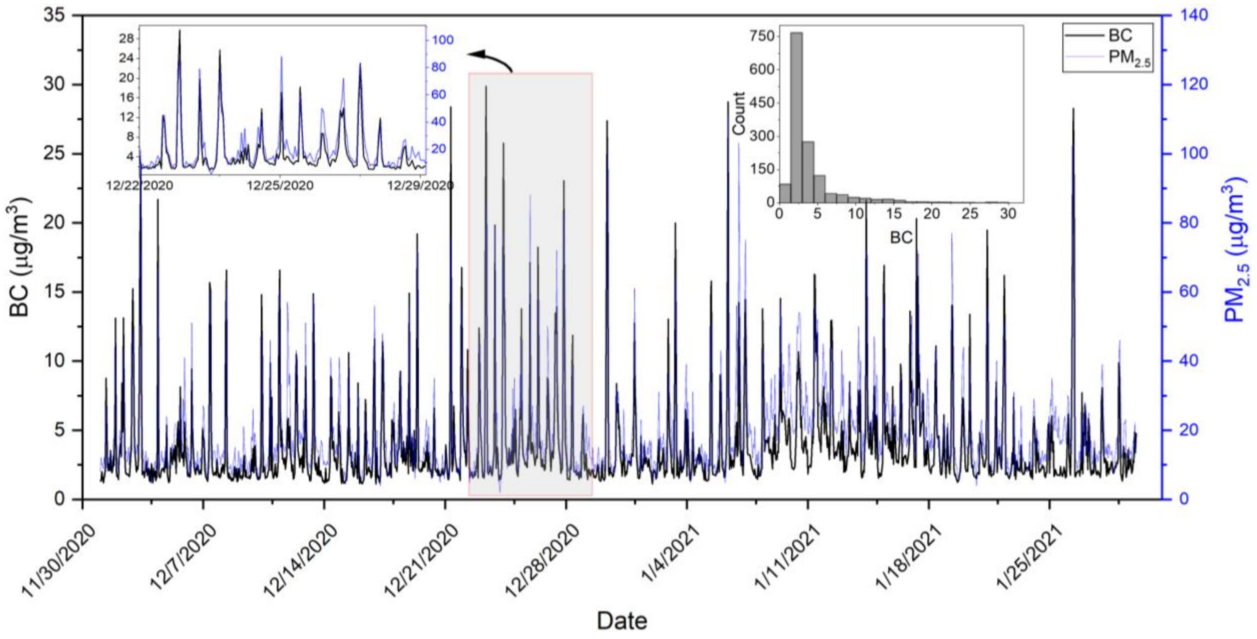

Figure S5: Hourly BC and $PM_{2.5}$ timeseries for Addis Ababa Central between December 1, 2020 – January 30, 2021. The timeseries inset plot shows a week's data in the shaded gray box. The histogram inset plot shows the distribution of BC concentrations for the Addis Ababa Central site during the sampling period.

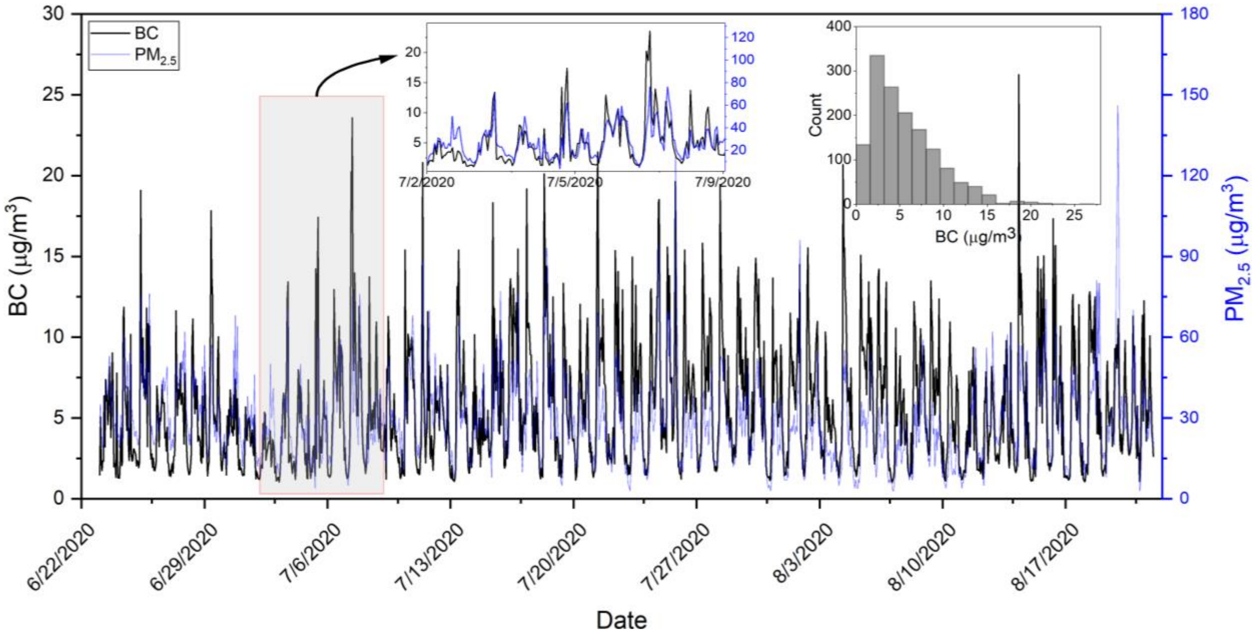

Figure S6: Hourly BC and $PM_{2.5}$ timeseries for Addis Ababa Central between June 23 – August 22, 2020. The timeseries inset plot shows a week's data in the shaded gray box. The histogram inset plot shows the distribution of BC concentrations for the Addis Ababa Central site during the sampling period.

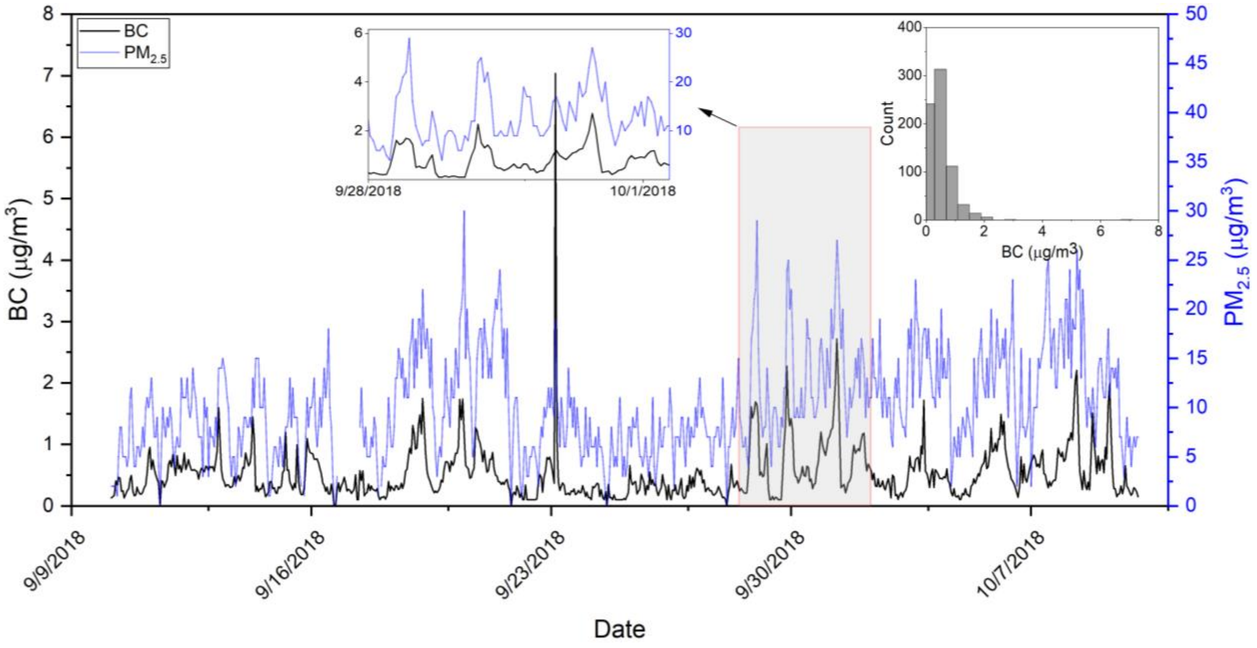

Figure S7: Hourly BC and $PM_{2.5}$ timeseries for Lawrenceville site between September 10 – October 10, 2018. The timeseries inset plot shows a week's data in the shaded gray box. The histogram inset plot shows the distribution of BC concentrations for Lawrenceville during the sampling period.

S7. BC:$PM_{2.5}$ timeseries at the measurement sites

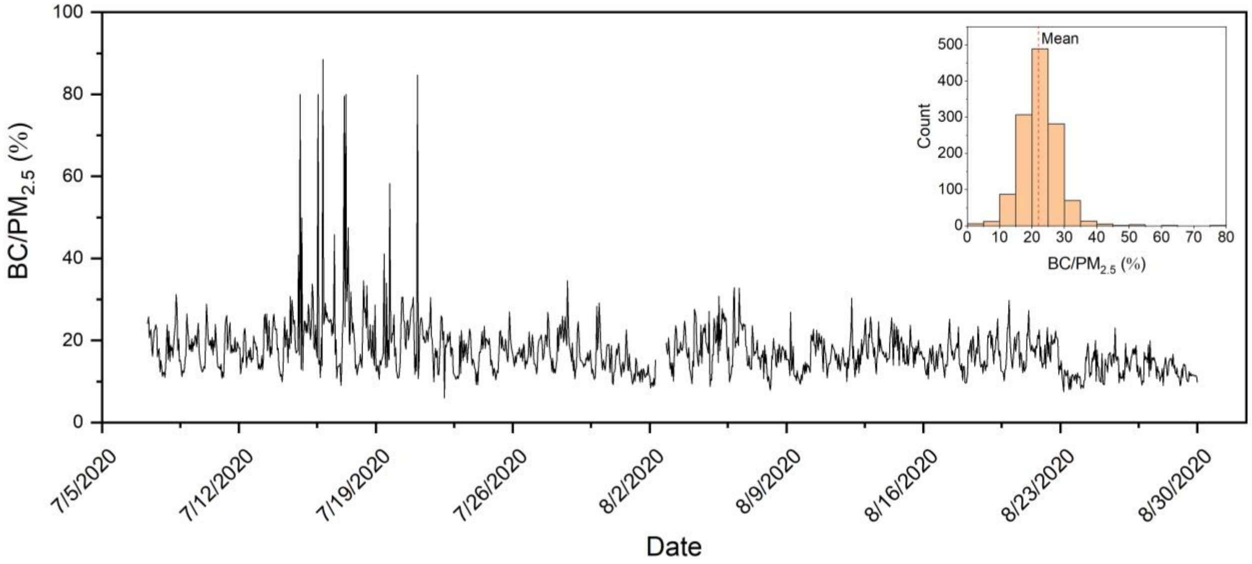

Figure S8: Hourly BC to $PM_{2.5}$ ratio (BC:$PM_{2.5}$ or BC/$PM_{2.5}$) timeseries for the Abidjan site. The inset histogram plot shows the distribution of BC:$PM_{2.5}$ and the vertical dotted line represents the mean ratio for the measurement period.

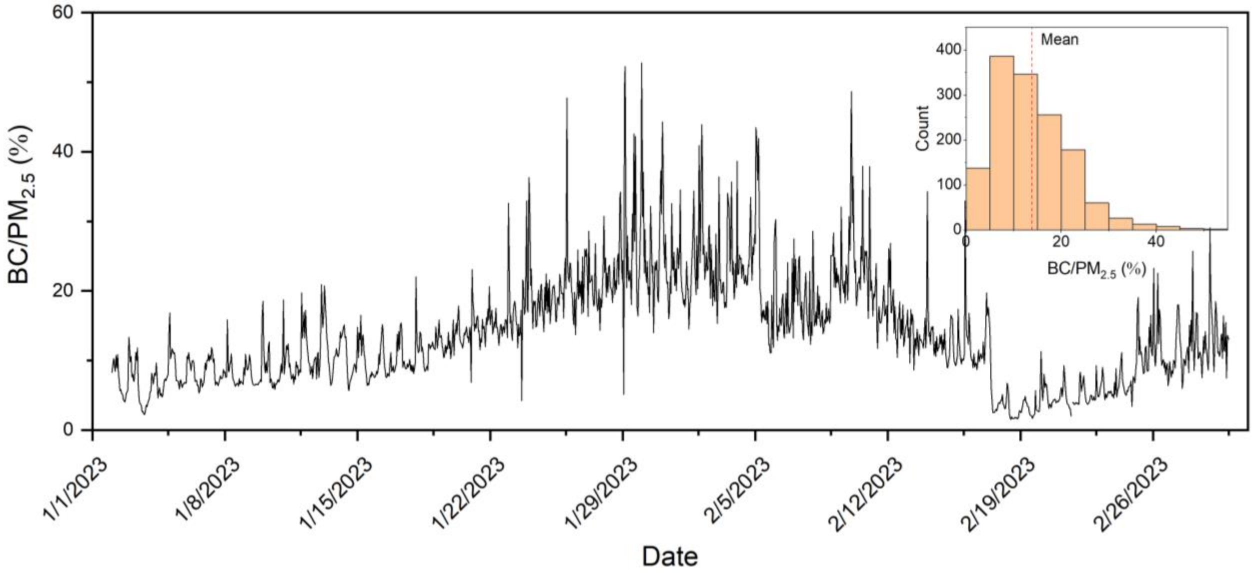

Figure S9: Hourly BC to $PM_{2.5}$ ratio (BC:$PM_{2.5}$ or BC/$PM_{2.5}$) timeseries for the Accra site. The inset histogram plot shows the distribution of BC:$PM_{2.5}$ and the vertical dotted line represents the mean ratio for the measurement period.

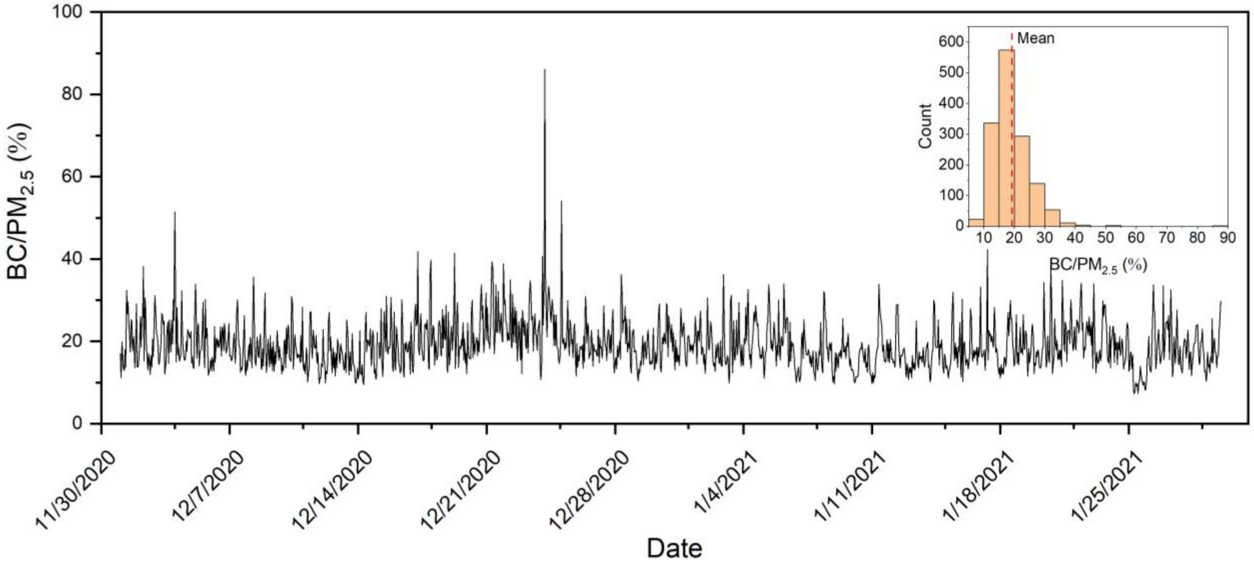

Figure S10: Hourly BC to $PM_{2.5}$ ratio (BC:$PM_{2.5}$ or BC/$PM_{2.5}$) timeseries for the Addis Ababa Central site during December 1, 2020 – January 30, 2021. The inset histogram plot shows the distribution of BC:$PM_{2.5}$ and the vertical dotted line represents the mean ratio for the measurement period.

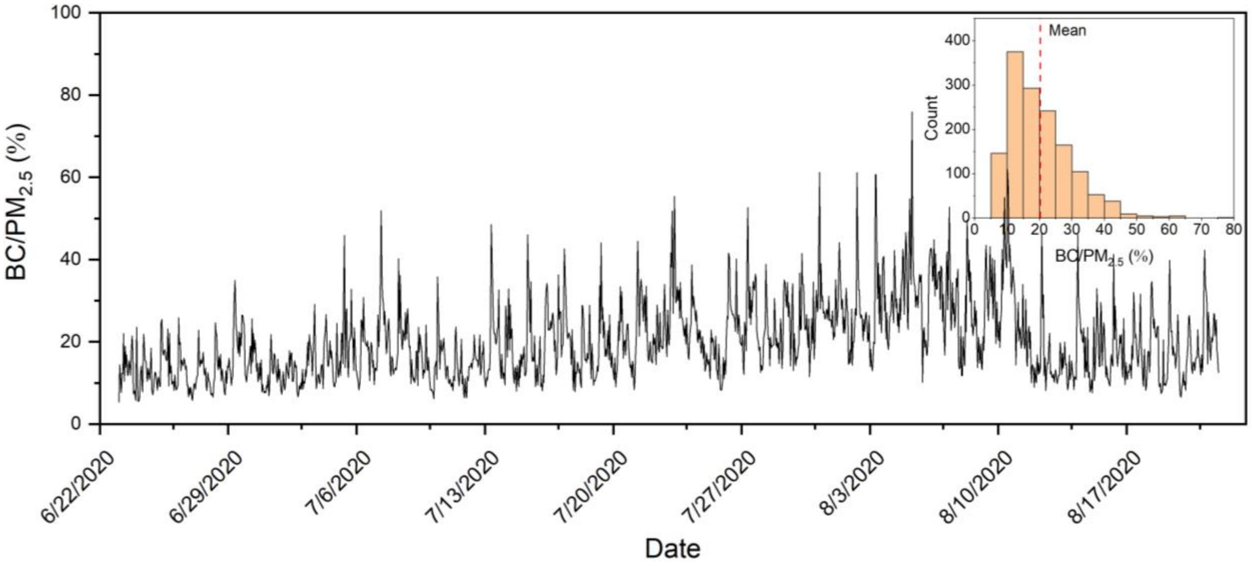

Figure S11: Hourly BC to $PM_{2.5}$ ratio ($BC:PM_{2.5}$ or $BC/PM_{2.5}$) timeseries for the Addis Ababa Central site during June 23 – August 22, 2020. The inset histogram plot shows the distribution of $BC:PM_{2.5}$ and the vertical dotted line represents the mean ratio for the measurement period.

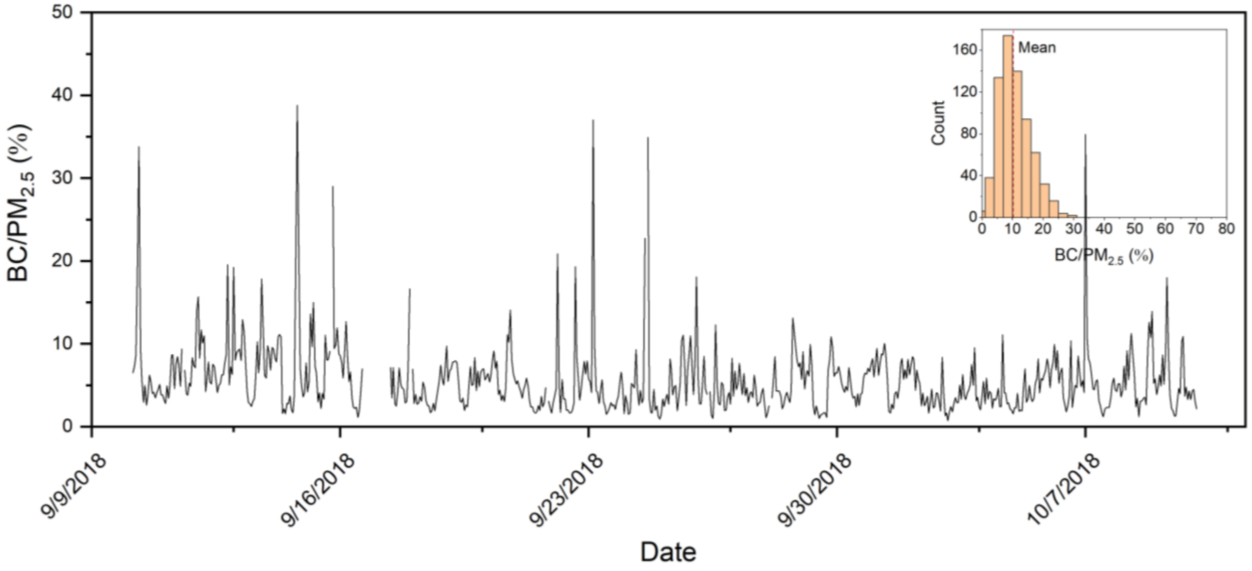

Figure S12: Hourly BC to $PM_{2.5}$ ratio ($BC:PM_{2.5}$ or $BC/PM_{2.5}$) timeseries for the Lawrenceville site during September 10 – August 10, 2020. The inset histogram plot shows the distribution of $BC:PM_{2.5}$ and the vertical dotted line represents the mean ratio for the measurement period.

S8. Low and high PM event comparison of BC, $PM_{2.5}$ and BC:$PM_{2.5}$ for Accra

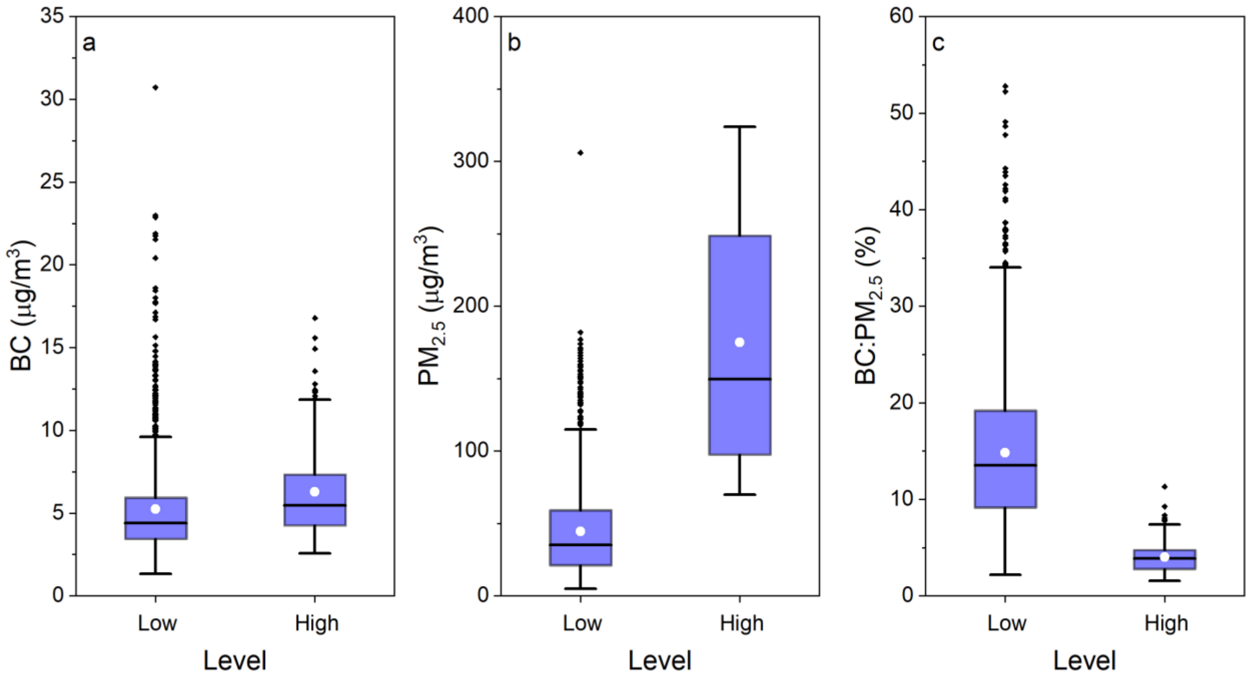

Figure S13: Box plot comparison of hourly BC, $PM_{2.5}$ and BC:$PM_{2.5}$ levels in 'Low' and 'High' PM events at Accra in 2023. High dust event (N = 125) corresponds to measurements during February 17 – 21, 2023 and the Low dust evet (N = 1291) includes measurements in the rest of the sampling period. The boxes show the interquartile range and whiskers on the two ends extend to 1.5 times the interquartile range. Horizontal black line inside the boxes are medians, white dots in the boxes represent mean values, and dots outside the whiskers are outliers.

Table S3: Mean hourly BC, $PM_{2.5}$ and BC:$PM_{2.5}$ for the 'Low' and 'High' dust events in Accra

**Pollutant Level**	R^2	**Mean BC (μg/m³)**	**Mean $PM_{2.5}$ (μg/m³)**	**Mean BC:$PM_{2.5}$ (%)**
High (N=125)	0.23	6.3 ± 2.9	175.1 ± 80.4	4.0 ± 1.8
Low (N=1291)	0.34	5.2 ± 2.9	44.5 ± 32.7	14.8 ± 7.5

S9. Seasonal comparison of BC, $PM_{2.5}$ and BC:$PM_{2.5}$ for Abidjan

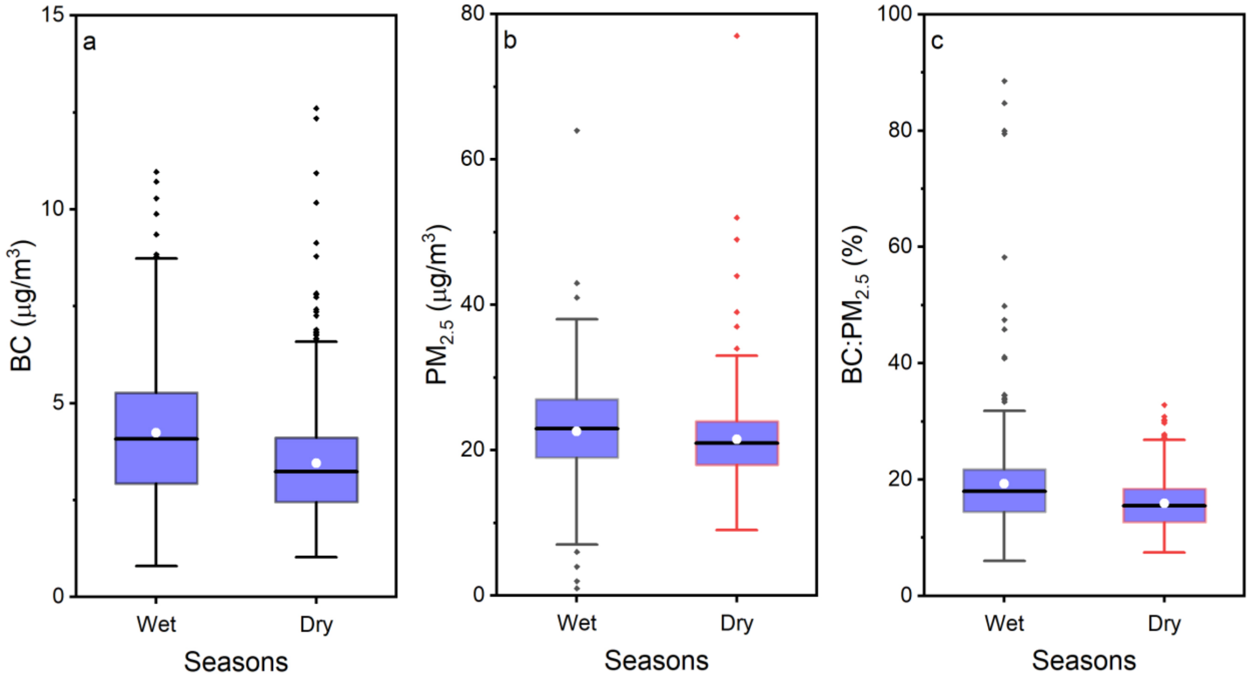

Figure S14: Box plot comparison of hourly BC, $PM_{2.5}$ and BC:$PM_{2.5}$ levels in wet and dry seasons at Abidjan in 2020. Wet season (N = 695) corresponds to measurements in July, 2020 and the dry season (N = 697) includes measurements in August of 2020. The boxes show the interquartile range and whiskers on the two ends extend to 1.5 times the interquartile range. Horizontal black line inside the boxes are medians, white dots in the boxes represent mean values, and dots outside the whiskers are outliers.

Table S4: Mean weather [3] and pollution parameters and for Wet and Dry seasons in Abidjan.

**Seasons**	**Mean Rainfall (mm)**	**Mean Wind Speed, W_S (km/h)**	**Mean Temp, T_S (°C)**	**Mean BC (µg/m³)**	**Mean $PM_{2.5}$ (µg/m³)**	**Mean BC:$PM_{2.5}$ (%)**
Wet (July)	5.4	18.2	24.7	4.2 ± 1.7	22.6 ± 6.5	19.2 ± 8.6
Dry (August)	1.2	16.9	24.0	3.4 ± 1.4	21.5 ± 5.5	15.9 ± 4.3

S10. Analyzing variations in diurnal trend across different days of the week

To test if the diurnal trends of black carbon (BC) for weekdays (Monday to Friday), Saturdays, and Sundays are distinct across different sites, we employed the Mann-Whitney U test, a non-parametric test suitable for small sample sizes and independent groups, unlike popular parametric tests, like, t-test and Analysis of Variance (ANOVA). This test does not assume normality, making it robust for our dataset of 24 mean values per category. We conducted pairwise comparisons between the day types to identify any significant differences in their distributions. The results are presented as U-statistics and p-values, with a significance level set at 0.05. This approach enabled us to effectively assess the diurnal trends of BC concentrations across various urban environments, ensuring that our conclusions were statistically sound despite the limited sample size and the non-normal distribution of the data. This test compares means across different time points (hours of a day) and conditions (weekdays, Saturdays, and Sundays). Therefore, the null hypothesis and alternate hypothesis can be states as follows.

Null hypothesis (H_0): There is no significant difference in the diurnal trends of BC levels among different day categories for a target site.

Alternate hypothesis (H_1): There is a significant difference in the diurnal trends of BC levels among different day categories for a target site.

We performed the significance tests at a 95% confidence level. If the p-value is greater than 0.05, we fail to reject the null hypothesis, indicating that there is no significant difference in the diurnal trends of black carbon (BC) levels between weekdays, Saturdays, and Sundays across different sites. Conversely, if the p-value is less than or equal to 0.05, we reject the null hypothesis,

suggesting significant differences in the diurnal trends of BC levels between the different day types and/or sites.

Table S5: Mann-Whitney U test summary to identify significant difference in diurnal trend of BC between weekday, Saturday and Sunday.

Site	Comparison	p-value	Significant difference
Abidjan, Cote D'Ivoire	Weekday – Saturday	0.327	No
	Weekday – Sunday	0.056	No
	Saturday – Sunday	0.135	No
Accra, Ghana	Weekday – Saturday	0.348	No
	Weekday – Sunday	0.392	No
	Saturday – Sunday	0.975	No
Addis Ababa Central site, Ethiopia - Winter (AAC-W)	Weekday – Saturday	0.37	No
	Weekday – Sunday	0.37	No
	Saturday – Sunday	1	No
Addis Ababa Central site, Ethiopia - Summer (AAC-S)	Weekday – Saturday	0.813	No
	Weekday – Sunday	0.992	No
	Saturday – Sunday	0.765	No
Addis Ababa Jacros site, Ethiopia – Winter (AAJ-W)	Weekday – Saturday	0.298	No
	Weekday – Sunday	0.252	No
	Saturday – Sunday	0.942	No

Lawrenceville, Pittsburgh, PA, USA	Weekday – Saturday	0.91	No
	Weekday – Sunday	0.959	No
	Saturday – Sunday	0.877	No

S11. Metrics description

Normalized room mean square error (NRMSE)

The normalized mean square error is calculated as:

$$\text{NRMSE} = \frac{\sqrt{\frac{1}{N}\sum_{i=1}^{N}(P_i - O_i)^2}}{\frac{1}{N}\sum_{i=1}^{N} O_i}$$

Where:

- P_i represents the predicted value for the ith observation,
- O_i represents the observed (or actual) value for the ith observation,
- N is the total number of observations.

Normalized mean bias (NMBE)

The formula for normalized mean bias error with respect to the mean observed value is given by:

$$\text{NMBE} = \frac{\frac{1}{N}\sum_{i=1}^{N}(P_i - O_i)}{\frac{1}{N}\sum_{i=1}^{N} O_i}$$

Where:

- P_i represents the predicted value for the ith observation,
- O_i represents the observed (or actual) value for the ith observation,
- N is the total number of observations.

Normalized mean absolute error (NMAE)

The formula for normalized mean absolute error with respect to the mean observed value is given by:

$$\text{NMAE} = \frac{\frac{1}{N}\sum_{i=1}^{N}|P_i - O_i|}{\frac{1}{N}\sum_{i=1}^{N} O_i}$$

- P_i represents the predicted value for the ith observation,
- O_i represents the observed (actual) value for the ith observation,
- N is the total number of observations, and
- $|P_i - O_i|$ is the absolute error for the ith observation

S12. Extraction of GEOS-CF data for the sites

Table S6: Site locations, their coordinates, and coordinates for the mid-point of the corresponding GEOS-CF grid cell.

Site location	Location coordinates	GEOS-CF grid center coordinate
Abidjan, Côte d'Ivoire (ABJ)	5.335040, -3.976045	5.25, -4.0
Accra, Ghana (ACC)	5.579480, -0.170623	5.5, -0.25
Addis Ababa Central, Ethiopia (AAC)	9.058586, 38.760151	9.0, 38.75
Addis Ababa Jacros, Ethiopia (AAJ)	9.011387, 38.820928	9.0, 38.75
Lawrenceville, Pittsburgh, USA (PIT)	40.465420, -79.960757	40.5, -80.0

Table S7: Statistical metrics to compare hourly ground measurements with GEOS-CF estimates BC, $PM_{2.5}$ and BC:$PM_{2.5}$. R^2, slope, NRMSE, NMBE and NMAE are unitless quantities normalized by mean, whereas mean hourly BC and $PM_{2.5}$ are reported in μg/m^3. BC:$PM_{2.5}$ values are in %.

Location	Pollutant	μ (Ground)	μ (GEOS)	R^2	Slope (BC-$PM_{2.5}$)	NRMSE	NMBE	NMAE
Abidjan	BC	3.81	0.75	0.72	0.17	0.91	-0.8	0.8
	$PM_{2.5}$	22.01	13.24	0.87	0.56	0.52	-0.4	0.45
	BC:$PM_{2.5}$	17.46%	5.67%	0.86	0.28	0.79	-0.68	0.68
Accra	BC	5.33	1.43	0.78	0.23	0.9	-0.73	0.73
	$PM_{2.5}$	55.98	58.76	0.87	0.86	0.5	0.05	0.35
	BC:$PM_{2.5}$	13.87%	2.91%	0.86	0.18	0.95	-0.79	0.79
AAC – W	BC	3.89	1.38	0.42	0.20	1.14	-0.65	0.69
	$PM_{2.5}$	19.79	18.25	0.62	0.67	0.75	-0.08	0.5
	BC:$PM_{2.5}$	19.18%	7.53%	0.80	0.36	0.69	-0.61	0.61
AAC – S	BC	5.63	1.75	0.34	0.18	1.03	-0.69	0.78
	$PM_{2.5}$	28.9	29.25	0.56	0.75	0.81	0.01	0.62
	BC:$PM_{2.5}$	20.23%	6.46%	0.72	0.27	0.83	-0.68	0.68
Lawrenceville	BC	0.53	0.89	0.69	0.62	1.15	0.67	0.87
	$PM_{2.5}$	10.38	23.82	0.77	0.73	1.65	1.29	1.32
	BC:$PM_{2.5}$	5.64%	3.94%	0.62	0.61	0.82	-0.3	0.5

REFERENCES

(1) *US Embassies and Consulates | AirNow.gov*. https://www.airnow.gov/international/us-embassies-and-consulates/ (accessed 2024-01-29).

(2) *Download Files | AirData | US EPA*. https://aqs.epa.gov/aqsweb/airdata/download_files.html (accessed 2024-01-29).

(3) *Global Historical Weather and Climate Data | Weather and Climate*. https://weatherandclimate.com/ (accessed 2024-01-28).
